# Effectiveness and cost-effectiveness of reactive, targeted indoor residual spraying for malaria control in low-transmission settings: a cluster-randomised, non-inferiority trial in South Africa

**DOI:** 10.1016/S0140-6736(21)00251-8

**Published:** 2021-02-27

**Authors:** David Bath, Jackie Cook, John Govere, Phillemon Mathebula, Natashia Morris, Khumbulani Hlongwana, Jaishree Raman, Ishen Seocharan, Alpheus Zitha, Matimba Zitha, Aaron Mabuza, Frans Mbokazi, Elliot Machaba, Erik Mabunda, Eunice Jamesboy, Joseph Biggs, Chris Drakeley, Devanand Moonasar, Rajendra Maharaj, Maureen Coetzee, Catherine Pitt, Immo Kleinschmidt

**Affiliations:** a Department of Global Health and Development, London School of Hygiene & Tropical Medicine, London, UK; b Department of Health Services Research and Policy, London School of Hygiene & Tropical Medicine, London, UK; c Department of Infectious Disease Epidemiology, London School of Hygiene & Tropical Medicine, London, UK; d Department of Infection Biology, London School of Hygiene & Tropical Medicine, London, UK; e Wits Research Institute for Malaria, School of Pathology, Faculty of Health Sciences, University of the Witwatersrand, Johannesburg, South Africa; f Health GIS Centre, South African Medical Research Council, Durban, South Africa; g Biostatistics Unit, South African Medical Research Council, Durban, South Africa; h Office of Malaria Research, South African Medical Research Council, Durban, South Africa; i School of Nursing and Public Health, University of KwaZulu-Natal, Durban, South Africa; j Centre for Emerging, Zoonotic and Parasitic Diseases, National Institute for Communicable Diseases, National Health Laboratory Service, Johannesburg, South Africa; k Institute for Sustainable Malaria Control, University of Pretoria, Pretoria, South Africa; l School of Health Systems and Public Health, University of Pretoria, Pretoria, South Africa; m Mpumalanga Provincial Malaria Control Programme, Nelspruit, South Africa; n Limpopo Provincial Malaria Control Programme, Polokwane, South Africa; o South Africa National Malaria Programme, National Department of Health, Pretoria, South Africa; p Southern African Development Community Malaria Elimination Eight Secretariat, Windhoek, Namibia

## Abstract

**Background:**

Increasing insecticide costs and constrained malaria budgets could make universal vector control strategies, such as indoor residual spraying (IRS), unsustainable in low-transmission settings. We investigated the effectiveness and cost-effectiveness of a reactive, targeted IRS strategy.

**Methods:**

This cluster-randomised, open-label, non-inferiority trial compared reactive, targeted IRS with standard IRS practice in northeastern South Africa over two malaria seasons (2015–17). In standard IRS clusters, programme managers conducted annual mass spray campaigns prioritising areas using historical data, expert opinion, and other factors. In targeted IRS clusters, only houses of index cases (identified through passive surveillance) and their immediate neighbours were sprayed. The non-inferiority margin was 1 case per 1000 person-years. Health service costs of real-world implementation were modelled from primary and secondary data. Incremental costs per disability-adjusted life-year (DALY) were estimated and deterministic and probabilistic sensitivity analyses conducted. This study is registered with ClinicalTrials.gov, NCT02556242.

**Findings:**

Malaria incidence was 0·95 per 1000 person-years (95% CI 0·58 to 1·32) in the standard IRS group and 1·05 per 1000 person-years (0·72 to 1·38) in the targeted IRS group, corresponding to a rate difference of 0·10 per 1000 person-years (–0·38 to 0·59), demonstrating non-inferiority for targeted IRS (p<0·0001). Per additional DALY incurred, targeted IRS saved US$7845 (2902 to 64 907), giving a 94–98% probability that switching to targeted IRS would be cost-effective relative to plausible cost-effectiveness thresholds for South Africa ($2637 to $3557 per DALY averted). Depending on the threshold used, targeted IRS would remain cost-effective at incidences of less than 2·0–2·7 per 1000 person-years. Findings were robust to plausible variation in other parameters.

**Interpretation:**

Targeted IRS was non-inferior, safe, less costly, and cost-effective compared with standard IRS in this very-low-transmission setting. Saved resources could be reallocated to other malaria control and elimination activities.

**Funding:**

Joint Global Health Trials.

## Introduction

The mass scale-up of malaria control interventions in endemic countries has driven major reductions in malaria morbidity and mortality over the past two decades. From 2000 to 2019, malaria case incidence in the WHO African Region declined by 38%, from 363 to 225 per 1000 population at risk, and the malaria mortality rate decreased by 67%, from 121 to 40 deaths per 100 000 population at risk.^1^ Vector control had a pivotal role in this success and was responsible for an estimated 78% of malaria cases averted between 2000 and 2015.^2^

Over the past few years, however, progress has stalled and, in some areas, reversed.^1^ Several factors probably contributed towards this trend. Although funding for malaria control and elimination greatly increased between 2000 and 2010, it has plateaued since.^1^ Global malaria funding fell from $3·2 billion in 2017 to $3·0 billion in 2019, well below the estimated $5·6 billion required annually to remain on track towards the WHO global malaria strategy targets.^1^ Widespread and increasing insecticide resistance has compromised the effectiveness of low-cost, pyrethroid-based vector control.^3^, ^4^, ^5^ The substantially higher costs of vector control with new or repurposed insecticides to address resistance^6^, ^7^, ^8^ add considerably to budgetary challenges.

More efficient strategies are therefore required to reduce malaria transmission. Where transmission is already very low, universal application of mass interventions might be unwarranted and unsustainable. Modelling studies suggest that targeting interventions to areas where there is evidence of recent malaria transmission is less costly and might be similarly effective compared with blanket strategies.^9^, ^10^, ^11^ Evidence from randomised trials shows that adding reactive, targeted interventions to existing population-wide routine interventions can be effective in further reducing malaria in low-transmission settings.^12^, ^13^, ^14^ However, no study has investigated whether widespread deployment of malaria interventions, such as indoor residual spraying (IRS), can be safely replaced by reactive, targeted interventions.

**Research in context**

**Evidence before this study**

We searched PubMed on June 30, 2020, using the terms “vector control” AND “malaria” AND “reactive” AND “target*”, with no restriction on language or dates. The search identified three studies detailing work done in Zambia evaluating the use of reactive case detection, in addition to standard vector control. The search identified one study that evaluated reactive indoor residual spraying (IRS) using pirimiphosmethyl as an adjunct to pre-season blanket IRS in Namibia. This large cluster-randomised controlled trial found that reactive vector control (involving IRS of at least seven households within 500 m of an index household) resulted in lower malaria case incidence (adjusted rate ratio 0·48, 95% CI 0·16–0·80; p=0·002) compared with clusters that only received pre-season spraying. This study did not publish cost-effectiveness estimates, although it was mentioned that there are plans to publish this information. Empirical evidence on the effectiveness of targeted reactive vector control is therefore scarce and no published evidence is available regarding the cost-effectiveness of such strategies. There have been no previous studies that investigated whether routine blanket vector control can be safely replaced with reactive targeted vector control.

**Added value of this study**

We did a cluster-randomised trial over two malaria seasons (2015–17) in a very-low-transmission setting in northeastern South Africa to compare annual mass IRS campaigns with a reactive, targeted IRS strategy. We showed that reactive, targeted spraying was non-inferior to routine mass spraying on the basis of our prespecified margin of fewer than 1 additional case per 1000 person-years. We collected cost data during the trial and modelled the cost-effectiveness of switching from an untargeted to a targeted strategy. At the incidence observed in the trial (0·9 local cases per 1000 person-years), we found that a targeted strategy would have a 94–98% probability of being cost-effective, and would be cost-effective up to an incidence of 2·0–2·7 cases per 1000 person-years.

**Implications of all the available evidence**

Together with previous evidence, this study suggests that targeted IRS could be cautiously implemented as an alternative to annual IRS campaigns in areas with very low malaria transmission and strong surveillance systems. Doing so would enable scarce resources available for malaria control to be more effectively used for other life-saving activities, such as enhanced case detection or increased disease surveillance.

IRS is recommended as a primary malaria vector control method by WHO, has been adopted as policy by national malaria control programmes in 42 African countries,^1^ and has contributed substantially to reducing malaria in many countries, including South Africa.^15^, ^16^, ^17^, ^18^ IRS consists of the application of insecticide to the interior walls of houses at least once per year, usually by seasonal spray personnel. Training, organising, and supervising sprayers can be logistically challenging and result in poor quality insecticide application and inadequate spray coverage.^19^ More judicious deployment of IRS could therefore achieve comparable effectiveness at lower cost in low-transmission settings. We aimed to assess the non-inferiority, cost, and cost-effectiveness of reactive, targeted IRS compared with standard IRS.

## Methods

### Study design and clusters

We did a cluster-randomised, open-label, non-inferiority trial in a low-transmission area of northeastern South Africa. Malaria transmission is low in South Africa and confined to border districts of the three northeastern provinces of Mpumalanga, Limpopo, and KwaZulu-Natal. From 2010 to 2014, an annual mean of 8781 cases was recorded nationally,^1^ of which a high proportion were imported.^20^ The primary vector is *Anopheles arabiensis*,^21^ and *Plasmodium falciparum* accounts for nearly all cases.^1^ IRS has been the primary malaria control strategy in South Africa since 1945.^22^ At the time of our study, pyrethroid insecticides were used for painted surfaces and dichloro-diphenyl-trichloroethane (DDT) for the relatively small proportion of surfaces that are unpainted.^21^

Malaria is notifiable by legal statute in South Africa. In malaria risk areas, clinical guidelines require all febrile patients presenting at (public and private) health facilities be tested for malaria by rapid diagnostic tests (First Response Malaria Ag *P falciparum* HRP2 Test; Premier Medical Corporation; Mumbai, India) or microscopy. Uncomplicated malaria cases are treated with artemether-lumefantrine.^23^ In 2019, single low dose primaquine in addition to artemisinin-based combination treatment was introduced in South Africa to reduce onward transmission.^23^ Intravenous artesunate is used to treat severe malaria. Health workers classify cases as imported if the onset of symptoms occurred 7–30 days after travel to a malaria endemic area outside the country.^24^ All deaths of people diagnosed with malaria are investigated by a clinician affiliated with the provincial malaria control programmes.

**Figure 1:**
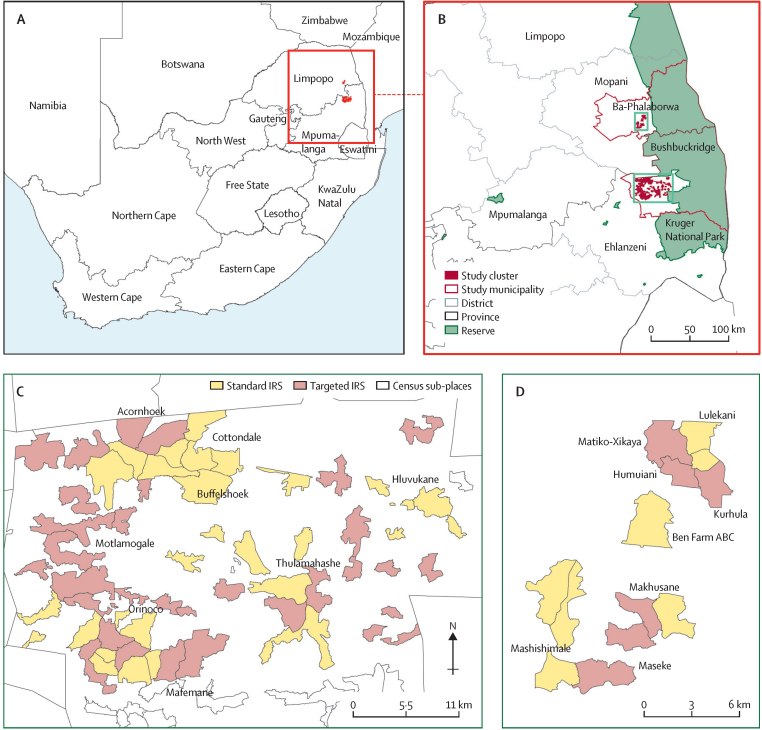
Study location (A) Location of trial provinces. (B) Location of clusters within trial provinces. (C) Allocation of clusters to study groups within Mpumalanga. (D) Allocation of clusters to study groups within Limpopo. IRS=indoor residual spraying.

This study took place between Aug 1, 2015, and July 31, 2017, in the predominantly rural subdistricts of Bushbuckridge in Mpumalanga province and Ba-Phalaborwa in Limpopo province (figure 1).^25^ In the study area, malaria incidence ranges between fewer than 1 case per 1000 person-years and 5 cases per 1000 person-years, and malaria is mostly locally acquired and seasonal between October and May.^20^, ^25^, ^26^ Neither pyrethroid nor DDT resistance have been reported in the trial provinces.^27^, ^28^

Census wards were mapped and formed into clusters comprising populations of about 5000–10 000 people. To be eligible for inclusion in the trial, clusters required a history of local cases in at least one year in the 5 years before the trial (Aug 1, 2010–July 31, 2015). Wherever possible, clusters were separated by natural boundaries or uninhabited space. Around 400 000 people resided within the trial clusters, the majority in Mpumalanga province (72%).

The trial's objective was to determine whether targeted IRS is non-inferior to the standard strategy, standard IRS, within a specified margin of non-inferiority, using passively reported malaria incidence as the primary outcome. A non-inferiority margin of 1 case per 1000 person-years was chosen; a greater difference in incidence between study groups would be important because South Africa's malaria elimination plan aimed to reduce incidence in all areas to below this threshold.

Ethics approvals were provided by institutional review boards at the London School of Hygiene & Tropical Medicine (7396-1), University of Witwatersrand (M140762), and Mpumalanga and Limpopo Provincial Departments of Health. An amendment detailing minor changes to the trial protocol was submitted on Oct 7, 2015 (appendix p 16), and ethics approval received on Dec 10, 2015. Community consent was sought through public meetings and discussions with ward councillors. During case investigation visits, written informed consent was obtained from householders. Independent trial steering and data safety monitoring committees oversaw the trial.

### Randomisation and masking

Clusters were randomly assigned (1:1) to receive either standard IRS or targeted IRS using restricted randomisation to balance study groups on pre-existing characteristics that might have been associated with the trial outcome. Restriction criteria were mean malaria incidence between Aug 1, 2010, and July 31, 2015 (obtained from the provincial reporting system), province, population size, proportion of households sprayed with IRS in 2014, population density, and total length of streams and rivers. The allocation of clusters to study groups was finalised at a randomisation ceremony attended by ward councillors. Blinding of communities or research personnel to study groups was not possible; however, data analysis was masked by labelling the groups as A and B.

### Procedures

The reference group, standard IRS, comprised the standard practice of annual mass spray campaigns by the provincial malaria control programmes, using either DDT or the pyrethroids deltamethrin (Bayer; Isando, South Africa) or α-cypermethrin (Efekto; Isando, South Africa). Spray training commenced in August, before the start of the malaria season, and spraying continued until December in both years. Following standard procedures, programme managers prioritised at-risk areas, such as those close to rivers and streams, on the basis of the number of malaria cases in the previous season, malaria control programme expert opinion, and available budget. Around a third of households in standard IRS clusters were sprayed annually through this informal targeting process. Spray operations were led by environmental health practitioners, who trained teams of seasonal contract sprayers over a 2-week period.

Confirmed malaria cases at health facilities are reported to the provincial malaria control programme within 24 h, triggering a case investigation.^29^ During the study, the provincial malaria control programme contacted the study coordinator to determine whether cases were in the study area and, if so, in which study group. In standard IRS clusters, case investigation teams were employed by the provincial malaria control programme. During an investigation, they administered a short questionnaire on recent travel history to determine whether the case was imported or locally acquired, following standard guidelines.^24^ If confirmed as locally acquired, the index case house was sprayed only if it had not already been sprayed during the annual spray round. Any household member reporting fever was tested using a malaria rapid diagnostic test and referred for treatment to the nearest health facility if positive.

In the intervention group, targeted IRS, no annual mass spray campaigns were done. Case investigations were triggered in the same way as in standard IRS clusters; however, case investigation teams were employed by the trial. In addition to case investigation activities, the teams sprayed the interiors of locally acquired index case houses and up to eight neighbouring houses (around 50 structures) within 200 m with deltamethrin, regardless of wall type, subject to consent of householders. DDT was considered unsuitable for targeted IRS, because most houses in the study area have painted surfaces. Malaria testing and referral was identical to standard IRS clusters. Further details of the interventions are provided in the appendix (pp 2–5).

To verify the quality of spray application, susceptible adult *A arabiensis* laboratory colony mosquitoes maintained at the Limpopo Malaria Control Programme insectary in Tzaneen were used for standard cone bioassay testing within 2–4 weeks of spraying in a random sample of households in the targeted IRS group.^30^, ^31^ Standard IRS was intended to represent standard practice, so no additional quality checks were done.

Case investigation data were collected on handheld tablet computers using an application (Mobenzi Researcher) developed for the trial, uploaded in real time using cellular network to a cloud-based server, and validated and analysed using Microsoft Excel 2016 and Stata version 15.

### Outcomes

The primary outcome, malaria incidence, was recorded through passive case detection at all health facilities within the subdistrict, as reported to the malaria control programme. Clinical malaria was diagnosed if the patient presented with a fever (axillary temperature ≥37·5°C) or history of fever (in the past 48 h), in the presence of parasitaemia confirmed by rapid diagnostic test or microscopy. Cases classified as imported were excluded from analysis. The trial data safety monitoring committee assessed reports of delays between diagnosis of cases and spraying in the targeted IRS group, and numbers of malaria cases and deaths between study groups to detect any unexpected increase in the targeted IRS group. Reports of malaria-associated deaths were submitted by the principal investigator to the data safety monitoring committee immediately after the provincial health department completed its investigation.

An endline cross-sectional survey was done from June 12 to Aug 16, 2017, to assess additional secondary outcomes, including household compliance, population attitudes to IRS, and serological markers. The proportion of structures targeted for IRS that were not sprayed and the reasons why structures were not sprayed, both secondary outcomes of the trial, were not consistently collected and hence not reported. Testing for insecticide resistance could not be done, because collection of sufficient mosquitoes for standard tests proved impossible.

### Statistical analysis

For sample size calculations, mean incidence of locally acquired malaria in the reference group was assumed to be 2·2 per 1000 person-years on the basis of historical data. Assuming a coefficient of variation between clusters of 0·5, the trial required 31 clusters per group (62 in total) of 6000 people each over 2 years (12 000 person-years per cluster) for 80% power at two-sided 5% (2·5% one-sided) significance to show non-inferiority within a margin of 1 case per 1000 person-years.^32^

The primary outcome, malaria incidence per cluster, was calculated from local cases recorded at health facilities and the cluster population recorded in the 2011 national census, projected forward. Cases were allocated to clusters on the basis of the place of residence provided by patients at the time of diagnosis and confirmed during case investigations. Incidence by group was calculated as the mean of the cluster incidences allowing for the clustered design. To assess non-inferiority, the incidence rate difference between study groups was estimated using linear regression on the cluster incidences with and without adjustment for province. Two-sided 95% CIs, corresponding to one-sided 97·5% intervals, and two-sided 90% CIs, corresponding to one-sided 95% intervals, were calculated as standard for non-inferiority tests. The upper limit of the CI of the difference in rates between study groups was compared with the prespecified margin of non-inferiority. p values indicate the probability of obtaining the given result by chance if the true rate difference is greater than the specified non-inferiority margin—ie, more than 1 case per 1000 person-years. Poisson regression adjusting for province was used to calculate rate ratios for cost-effectiveness analyses.^33^

### Cost-effectiveness analysis

Resource use and cost of each resource (ie, unit cost) were collected to model the real-world cost for the provincial malaria control programme to implement either standard IRS or targeted IRS as standard practice. Costs were estimated for each strategy using a combination of top-down and bottom-up costing.^34^ The focus was on economic costs, which reflect the full value of all resources used; however, almost all economic costs were also financial costs, meaning they involved monetary payment. A health services perspective was adopted, which included the costs of spraying, case investigations, training, supervision, diagnosis, and treatment; costs borne by households were excluded due to lack of data. Set-up costs for targeted IRS and recurrent costs for standard IRS and targeted IRS were estimated. Costs for the provincial malaria control programme to set up targeted IRS, including training, were annualised assuming a useful life of 3 years. Costs were estimated for Mpumalanga province only (where 49 of the 62 clusters were located), because detailed accounts from the Limpopo Malaria Control Programme were unavailable. Research costs were excluded.

Where implementation costs incurred during the trial were expected to differ from real-world implementation outside a trial, cost estimates were adjusted to best approximate real-world implementation. All resources were costed using unit costs from the Mpumalanga Malaria Control Programme, even where the trial obtained them (for targeted IRS) at a different unit cost. It was assumed that implementation of targeted IRS would not change malaria control programme management and overheads, and that environmental health practitioners leading the annual mass spray campaigns would remain employed under targeted IRS.

The cost per case diagnosed and treated was assumed to be the same under either strategy and estimated from available secondary data (appendix pp 6–8).^35^, ^36^, ^37^ Costs were estimated for a standardised population of 100 000 in constant 2017 US$.^38^ Details of costing methods are provided in the appendix (pp 9–13).

The incremental cost savings per disability-adjusted life-year (DALY) incurred by targeted IRS compared with standard IRS were calculated to determine whether switching to targeted IRS would be cost-effective. This formulation differs from (but is equivalent to) the typical presentation of incremental cost per DALY averted, because targeted IRS is designed to be less costly but potentially (slightly) less effective than its comparator. Incremental cost-effectiveness ratios (ICERs) were compared with the lowest ($2637, 43% of gross domestic product [GDP] per capita) and highest ($3557, 58% of GDP per capita) of four cost-effectiveness thresholds,^39^ which reflect the benefits forgone in withdrawing resources from an existing intervention in South Africa. Cost-effectiveness results were estimated overall (ie, for both years combined) and by year.

A decision tree was used to model costs and health outcomes over a lifetime horizon (appendix p 14). DALYs were modelled as the sum of years of life lost and years of life lived with disability using a discount rate of 3% and no age weighting (appendix p 15).^40^

The impact of uncertainty in individual input parameters on cost-effectiveness was explored through deterministic sensitivity analysis by varying parameters individually across plausible value ranges. Probabilistic sensitivity analysis was done to explore the combined impact of parameter uncertainty. Proportions were assumed to follow beta distributions; non-negative parameters, such as costs, were assumed to be gamma distributed. By use of Monte Carlo simulation, 10 000 samples were drawn from the parameter distributions and used to calculate incremental costs and effects, which were plotted on the cost-effectiveness plane alongside cost-effectiveness thresholds. Mean ICERs were calculated as the mean incremental cost across iterations divided by the mean incremental DALYs across iterations. 95% credible intervals were calculated as percentiles of the ICER distribution. Using cost-effectiveness acceptability curves,^41^ the probability that switching from standard to targeted IRS would be cost-effective at different thresholds was calculated.

Malaria incidence at which the more cost-effective strategy would change was calculated in a threshold analysis. For targeted IRS, it was assumed that the cost of insecticide, as well as diagnostic and treatment costs, would increase in proportion to the number of cases, but that other resources associated with case investigations (case investigators, equipment, and transport costs) would remain fixed. For standard IRS, only diagnostic and treatment costs were assumed to vary with the number of cases; all other resources would remain fixed. Incidence was varied until the ICER (for the 2 years combined) equalled each of the cost-effectiveness thresholds. Details of model parameters are provided in the appendix (pp 6–8).

This study is registered with ClinicalTrials.gov, NCT02556242.

### Role of the funding source

The funder of the study had no role in study design, data collection, data analysis, data interpretation, or writing of the report.

## Results

The study profile is provided in the appendix (p 17). Characteristics at baseline are summarised in table 1. During the 2-year trial period, 1030 malaria cases were recorded, of which 705 (68%) were classified as locally acquired, corresponding to a crude incidence of 0·90 local cases per 1000 person-years (table 2). In Limpopo, incidence was 1·88 cases per 1000 person-years and in Mpumalanga 0·65 cases per 1000 person-years. Annual incidence was 0·05 per 1000 person-years (n=20) in year 1 and 1·74 per 1000 person-years (n=685) in year 2. Incidence by cluster varied from zero cases (seven clusters, all in Mpumalanga: three in the standard IRS group and four in the targeted IRS group) to more than 3 cases per 1000 person-years (four clusters, of which three were in Limpopo: two in the standard IRS group and two in the targeted IRS group). Case incidence varied substantially over time, peaking in May, 2017, when 54% (n=383) of all locally acquired cases occurred (appendix p 18). Median age of individuals with locally acquired malaria was 27 years (IQR 10–37; range 0–91), and 373 (53%) of 705 cases occurred in men.

**Table 1:** Baseline characteristics

		**Standard IRS group**	**Targeted IRS group**
Number of clusters		31	31
	Mpumalanga	24	25
	Limpopo	7	6
Mean cluster population (SD)		6102 (3254)	6588 (2225)
Mean number of households per cluster (SD)		1534 (802)	1619 (545)
Mean population density per cluster (SD)		1095 (447)	1091 (510)
Mean annual local malaria case incidence per 1000 population for 2010–15 (SD)		1·05 (0·89)	0·88 (0·89)
Mean percentage of households sprayed by IRS in the previous year (SD)		41·4% (29·3)	38·7% (23·2)

IRS=indoor residual spraying.

**Table 2:** Local malaria cases, crude incidence, and cost, by year and study group

	**Standard IRS**	**Targeted IRS**	**Total**	**Standard–targeted rate difference (95% CI)**	**Incremental cost (% standard IRS)**
**Group size**					
Number of clusters	31	31	62	..	..
Study population	189 150	204 237	393 387	..	..
**Year 1**					
Number of local malaria cases	7	13	20	..	..
Number of deaths	1	0	1	..	..
Crude incidence per 1000 person-years	0·04	0·06	0·05	..	..
Total economic cost per 100 000 population, constant 2017 US$	$189 118	$85 432	..	..	−$103 685 (−55%)
Economic cost per structure sprayed, constant 2017 US$	$2·78	$737·86	..	..	$735·07 (26 409%)
**Year 2**					
Number of local malaria cases	304	381	685	..	..
Number of deaths	11	9	20	..	..
Crude incidence per 1000 person-years	1·64	1·94	1·74	..	..
Total economic cost per 100 000 population, constant 2017 US$	$178 861	$90 733	..	..	−$88 127 (−49%)
Economic cost per structure sprayed, constant 2017 US$	$3·75	$26·20	..	..	$22·45 (598%)
**Total**					
Number of local malaria cases	311	394	705	..	..
Number of deaths	12	9	21	..	..
Crude incidence per 1000 person-years	0·82	0·96	0·90	..	..
Malaria case incidence* per 1000 person-years (95% CI)	0·95 (0·58 to 1·32)	1·05 (0·72 to 1·38)	..	0·10 (−0·38 to 0·59)†	..
Total economic cost per 100 000 population, constant 2017 US$	$184 319	$88 258	..	..	−$96 061 (−52%)
Economic cost per structure sprayed, constant 2017 US$	$3·19	$49·23	..	..	$46·05 (1444%)

IRS=indoor residual spraying.

* Mean of cluster incidences.

† Non-inferiority p value <0·0001.

There were 21 deaths (12 in the standard IRS group and nine in the targeted IRS group) associated with locally acquired malaria during the study period, corresponding to a case fatality rate of 3·0% (table 2). The median age at death was 40 years (IQR 34–53; range 22–66). The most common factors contributing to death were late presentation (n=8) and patients not being tested for malaria at first presentation (n=5). No other serious adverse events were reported.

In Mpumalanga, 128 519 structures (broadly defined as individual rooms within a house or outbuilding) were sprayed in standard IRS clusters in the first year and 90 196 in the second; in targeted IRS clusters, 132 and 6163 structures were sprayed in the same periods. In Limpopo, 150 structures were sprayed in targeted IRS clusters in the first year and 1622 in the second; data on the number of structures sprayed in standard IRS clusters were not available for Limpopo (appendix p 17). In the targeted IRS group, teams sprayed on average 3·7 neighbouring houses per investigation, despite repeat visits. Case investigation teams reported that, due to scattered settlement patterns, index case houses often had fewer than eight houses within 200 m. Cone bioassay testing done on 14 sprayed structures in the targeted IRS group reported an overall 24-h mosquito mortality of 99·5% (n=420), providing assurance of satisfactory insecticide application. Results of the endline survey are reported in the appendix (p 19).

**Figure 2:**
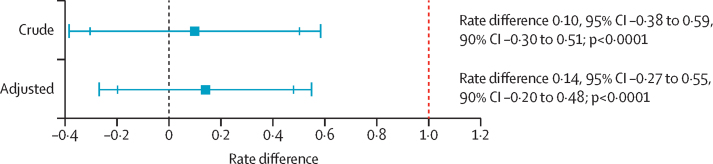
Rate difference between targeted IRS and standard IRS for 2-year study period This figure shows the rate difference between annual cluster incidence, crude and adjusted for province, with large caps representing 95% CIs and smaller caps representing 90% CIs. The red dotted vertical line represents the non-inferiority margin (1 case per 1000 person-years increase in incidence). Non-inferiority p value is the probability of obtaining the rate difference by chance if the actual difference is more than 1. Margin of non-inferiority is breached if CIs encompass 1. IRS=indoor residual spraying.

Malaria case incidence was 0·95 per 1000 person-years (95% CI 0·58 to 1·32; n=311) in the standard IRS group and 1·05 per 1000 person-years (95% CI 0·72 to 1·38; n=394) in the targeted IRS group (table 2); the rate difference was 0·10 (95% CI −0·38 to 0·59), equivalent to one extra case for every 10 000 people in the targeted IRS group (figure 2). There was strong evidence of non-inferiority of targeted IRS compared with standard IRS within the predefined margin of 1 case per 1000 person-years at the 2·5% one-sided significance level (p<0·0001). Adjusted for province, the rate difference was 0·14 (95% CI −0·27 to 0·55; figure 2). Further sensitivity analysis is reported in the appendix (p 20). In year 1, there was strong evidence that targeted IRS was non-inferior to standard IRS (adjusted rate difference 0·04, 95% CI −0·07 to 0·16; p<0·0001). For year 2, the 95% CIs crossed the non-inferiority margin for both the crude and adjusted rate differences, but the 90% CI (equivalent to 5% one-sided significance level) for both estimates remained below the non-inferiority threshold (adjusted rate difference 0·24, 95% CI −0·54 to 1·03, 90% CI −0·41 to 0·90; p=0·058; appendix p 21).

The average annual economic cost was $88 258 per 100 000 population for targeted IRS, 52% less than standard IRS ($184 319; table 2). Targeted IRS cost less, because it involved spraying fewer structures (around 3% of the structures sprayed in standard IRS), it did not use contract sprayers, and it used substantially less insecticide, transport, and equipment. Except for contract sprayers, personnel costs were similar across the two groups. Malaria treatment costs comprised a very small proportion of total costs under standard (0·9%) and targeted (2·3%) IRS. Further results by cost component are reported in the appendix (p 22).

**Figure 3:**
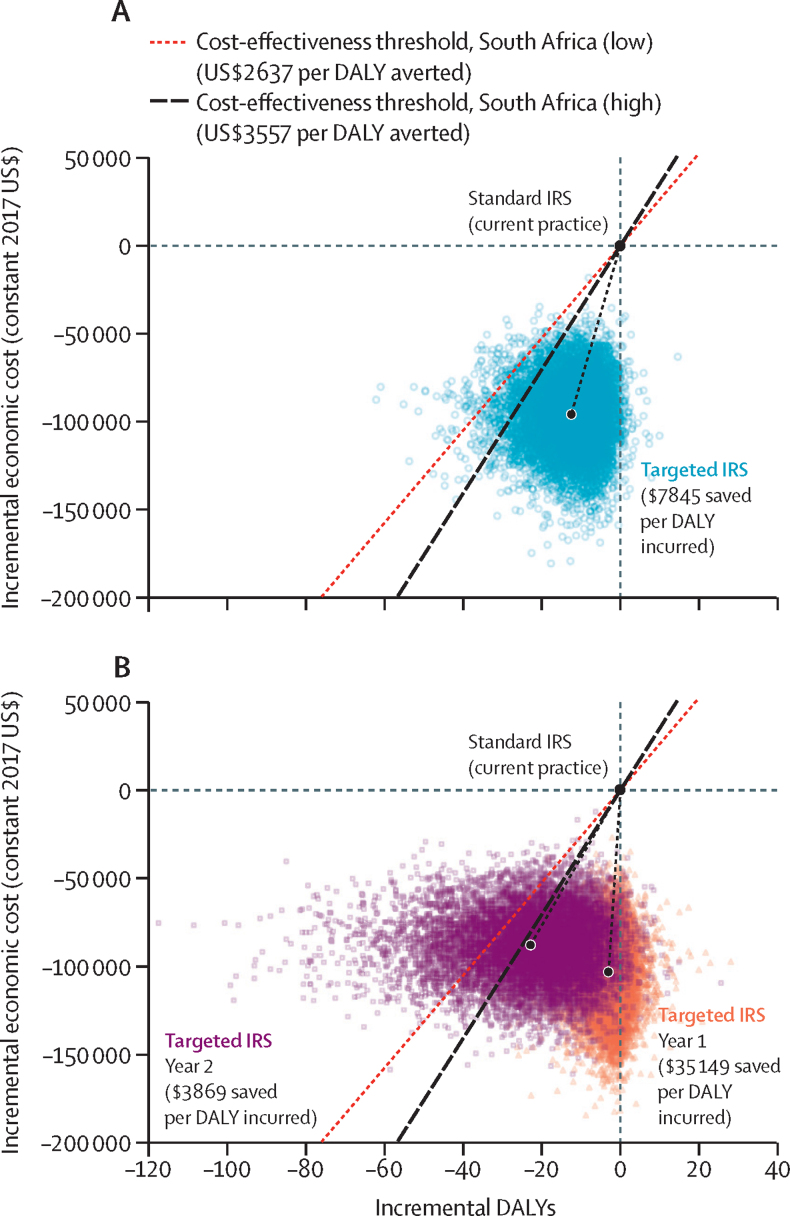
Cost-effectiveness plane Economic cost savings (from a health service perspective) and DALYs incurred by switching from standard IRS to targeted IRS are shown for the 2-year trial period (A) and individual study years (B). Costs and DALYs shown in the figure are incremental with respect to standard IRS (which is shown at [0,0]) in each study year. The large dots show the mean incremental cost and mean incremental DALYs across the 10 000 model simulations. Individual model simulations are shown as smaller dots, for each year. DALY=disability-adjusted life-year. IRS=indoor residual spraying.

Across the 2-year trial, targeted IRS saved $7845 (95% CI 2902–64 907) for each additional DALY incurred relative to standard IRS (figure 3). In year 1, when incidence was lower, targeted IRS saved $35 149 for each additional DALY incurred; the lower bound of the 95% CI was $6481 and at the higher bound targeted IRS was dominant—ie, less expensive and more effective than standard IRS. In year 2, when incidence was higher, targeted IRS saved $3869 (95% CI $1371–$50 689) per DALY incurred. At both cost-effectiveness thresholds ($2637 and $3557), targeted IRS would be considered cost-effective across the trial period and in each of the 2 years (figure 3)—ie, the cost savings from switching from standard to targeted IRS would be expected to generate greater net health benefits if invested elsewhere in the health system. At the incidence observed in the trial, targeted IRS would have a 94–98% probability of being the cost-effective choice at either cost-effectiveness threshold (figure 3; appendix p 23).

**Figure 4:**
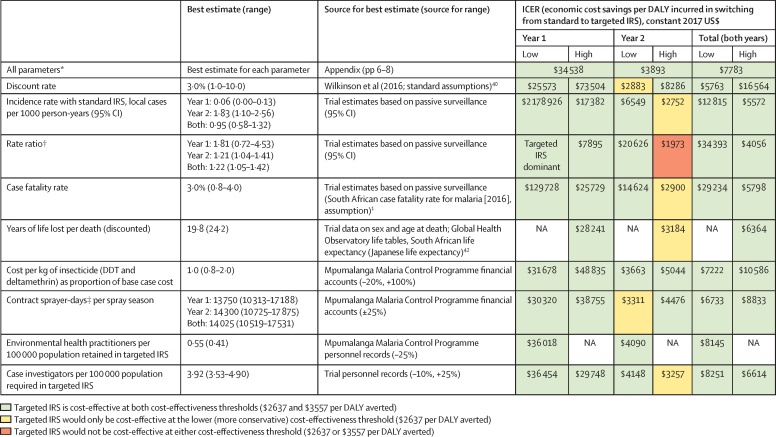
Deterministic sensitivity of ICERs to plausible variation in individual model parameters Where targeted IRS is the dominant strategy (ie, less costly and more effective than standard IRS), this has been stated in the cell. DALY=disability-adjusted life-year. ICER=incremental cost-effectiveness ratio. IRS=indoor residual spraying. NA=not applicable. *ICER estimates are based on best estimates for each parameter; therefore, they slightly differ from the ICER estimates presented in the main text, which are calculated as the mean incremental cost savings divided by the mean incremental DALYs from 10 000 model simulations. † Adjusted for province. ‡Contract sprayer-days is the number of contract sprayers employed each year multiplied by the number of days in the annual mass spraying season.

The finding that targeted IRS was cost-effective across the 2-year trial period is robust to plausible variation in all individual parameters evaluated (figure 4) and to re-analysis on the basis of Mpumalanga-specific incidence and rate ratios (appendix p 25). Year 1 results are similarly robust. If implemented in year 2 only, when incidence was higher, targeted IRS would not be cost-effective with respect to either of the cost-effectiveness thresholds if the rate ratio were at the upper bound of the 95% CI; targeted IRS would be cost-effective at the lower but not the higher threshold for plausible variation in six of the eight other parameters explored (figure 4). If all parameters except incidence remained constant, we estimated that targeted IRS would remain the preferred strategy up to an incidence of 2·0–2·7 cases per 1000 person-years, using the higher and the lower cost-effectiveness thresholds.

## Discussion

This study has shown that reactive, targeted IRS in response to index cases was non-inferior to mass annual IRS campaigns, which prioritise areas selected on the basis of historical information and expert opinion, within a margin of 1 case per 1000 person-years in this low-transmission setting in South Africa. In this context, changing from standard to targeted IRS would be a more efficient use of scarce malaria control programme resources, while providing non-inferior malaria protection. By adopting targeted IRS, the malaria control programme would make substantial savings, which could be redirected to other, potentially more efficient and life-saving malaria interventions, such as improved awareness of malaria risk among affected communities and health-care providers, enhanced case management, surveillance, and border screening. If targeted IRS were standard practice in this setting, adoption of standard IRS (at an ICER of $7845 per DALY averted) would not be considered cost-effective. However, replacing the existing standard IRS intervention that has been in place for many years would be politically and socially sensitive^43^ and would require caution and vigilance to avoid resurgence in cases.

These findings were generally robust to plausible changes in key parameters and are conservative in several ways. Cost savings would have been greater if we had included the substantial increases in insecticide costs that are expected when insecticide resistance necessitates a switch to more expensive next generation insecticides.^5^, ^44^ Although South Africa's overall malaria case fatality rate was 0·79% in the first study year and 1·06% in the second,^1^ our cost-effectiveness estimates were based on the observed study case fatality rate of 3·0%. Although mortality was similar in the two study groups, at lower overall case fatality rates, targeted IRS would be more cost-effective because any additional cases (relative to standard IRS) would result in fewer deaths and DALYs. Targeted IRS was much more cost-effective in year 1 than year 2, due to substantial between-year heterogeneity in incidence; however, targeted IRS remained the more cost-effective strategy (61–78% probability) in year 2 (appendix p 23).

In South Africa, malaria incidence often varies substantially between years.^1^, ^22^ Incidence was markedly higher during the second year of this study than the first.^1^ In standard IRS clusters, fewer structures were sprayed in year 2 because the informal targeting is based partly on the (very low) malaria incidence in the preceding season. In targeted IRS clusters, more structures were sprayed in year 2 in accordance with the protocol. The increased incidence resulted in some delays to the reactive spraying, which might have caused onward transmission and an increase in case numbers. In year 2, targeted IRS remained non-inferior to standard IRS at the one-sided 5% significance level, but not at the 2·5% significance level. The finding of more marginal non-inferiority at higher incidence should be interpreted with caution, however, because the trial was not powered to demonstrate non-inferiority in individual study years.

Our study has several limitations. First, incidence was measured using passive case detection, so any asymptomatic infections that could lead to onward malaria transmission would remain undetected. In South Africa, however, the vast majority of cases are symptomatic because the population does not have partial immunity, due to low exposure to malaria parasites. Additionally, self-medication is unlikely because the informal sector does not sell antimalarials. We therefore consider passive case detection robust in this setting. Second, possible misclassification of local cases as imported might have reduced the impact of the targeted IRS strategy because reactive spraying was only done in response to locally acquired cases. Third, in the targeted IRS group, fewer than half of the intended eight neighbouring houses were sprayed on average. Although the intervention would be more expensive at higher coverage, it might also be more effective. Fourth, costs were only estimated for Mpumalanga province, as such information was not available from Limpopo; however, results are robust to wide variation in unit costs and resource use (figure 4). Fifth, we did not quantify the inconvenience or potential secondary benefits (such as insect control) of receiving IRS or the costs to households of experiencing and seeking treatment for malaria; however, we expect these factors to have a relatively small incremental impact. Finally, we assumed that real-world implementation of targeted IRS by the provincial malaria control programme would achieve similar effectiveness as the trial without additional management and supervision. We consider this assumption reasonable because malaria control programme managers (who would deliver targeted IRS in a future implementation) receive similar remuneration to the trial managers who delivered targeted IRS in the study, and because we conservatively assumed that staff managing the spray programme would be retained (appendix pp 9–13).

This trial is the first to directly compare reactive IRS with annual, mass IRS campaigns. Previous studies have evaluated the effect of adding reactive targeted interventions, including IRS and drug administration, to routine population-wide measures, to further reduce or eliminate malaria.^13^, ^14^, ^45^, ^46^, ^47^ A trial in Namibia showed effective reduction of malaria incidence through reactive focal mass drug administration and reactive focal IRS, alone and in combination, when added to standard IRS.^14^ Our trial is unique in using a non-inferiority design to assess whether replacing annual IRS campaigns with reactive IRS targeting at-risk neighbourhoods would be safe and prevent malaria from surging out of control. As governments seek more sustainable strategies to enable the best use of scarce resources, non-inferiority trials could become more commonplace.

We expect our findings to be generalisable to very-low-transmission settings with well functioning surveillance systems. The reference, standard IRS, already uses informal targeting, prioritising areas on the basis of the resources available, local information, and malaria incidence in the previous year. Because many countries have adopted similar targeting strategies,^48^, ^49^ our findings regarding a cost-effective, data-driven, alternative strategy are expected to be widely relevant. The reactive targeted strategy we evaluated relies on well functioning surveillance systems; such systems are considered essential by WHO for countries pursuing malaria elimination.^50^, ^51^ Our results showed that targeted IRS would cease to be cost-effective in South Africa above an incidence of 2·0–2·7 per 1000 person-years, depending on the cost-effectiveness threshold used. We would therefore only recommend this strategy when transmission is proven to be very low through reliable case reporting.

This study highlighted weaknesses in malaria control that would benefit from investment, which could be achieved with the savings obtained from targeted IRS. The observed case fatality rate was high and indicated the need for appropriate interventions. Despite good knowledge about malaria reported in a household survey,^52^ some of the deaths during the study period were attributed to late presentation to a health facility, which could reflect low care seeking in populations in which malaria is rare.^53^ Lack of malaria testing at first presentation was also stated on some death reports, and might be associated with stockouts of rapid diagnostic tests at health facilities that, although infrequent, occurred during the trial.^54^ Malaria deaths have declined in number in South Africa since 2017 in response to corrective action that has already been implemented at health facilities.^55^ Reallocation of resources to comprehensive awareness campaigns encouraging prompt facility visits in response to symptoms and routine testing for malaria even when cases are rare could reduce or eliminate malaria mortality. Enhanced resilience against malaria commodity stockouts would further reduce the potential for malaria deaths. Additional investment could reduce the time interval between case diagnosis and case investigation. To minimise onward transmission, case investigations need to occur promptly, ideally within 48 h of case detection. In this trial, case investigations were frequently delayed when cases were more numerous, consistent with previous findings in South Africa.^20^ Case reporting using mobile phones^56^, ^42^ has since been implemented and has helped improve response times. Sufficient standby capacity of case investigation teams in a targeted IRS strategy will be essential to ensure that health services are prepared for unexpected surges in cases.

The commitment by heads of states in the Southern African Development Community to eliminate malaria in the region by 2030^57^ is commendable and ambitious. Fulfilling this goal will require identification of the most cost-effective ways to allocate scarce resources in changing epidemiological contexts. The withdrawal of mass prevention efforts in an uncoordinated manner motivated solely by cost considerations would be irresponsible and risk a rebound in malaria. The strategy presented here, reactive targeting of IRS on the basis of evidence of recent transmission, was shown to be safe and highly cost-effective in a pre-elimination setting and could free up vital resources for other, life-saving malaria services. Its implementation should be cautiously considered in settings where malaria transmission is already very low, case surveillance is robust, and health systems are able to respond nimbly to resurgent outbreaks.

## Data sharing

Deidentified data will be made available upon reasonable request to the corresponding author.
