# Supplementary material for: Effectiveness and cost-effectiveness of reactive, targeted indoor residual spraying for malaria control in low-transmission settings: a cluster-randomised, non-inferiority trial in South Africa
Source: Lancet. 2021 Feb 27;397(10276):816–27. doi: 10.1016/S0140-6736(21)00251-8 (PMC7910276; doi:10.1016/S0140-6736(21)00251-8)
Supplement: Supplementary appendix [file mmc1.pdf]

THE LANCET

## Supplementary appendix

This appendix formed part of the original submission and has been peer reviewed. We post it as supplied by the authors.

Supplement to: Bath D, Cook J, Govere J, et al. Effectiveness and cost-effectiveness of reactive, targeted indoor residual spraying for malaria control in low-transmission settings: a cluster-randomised, non-inferiority trial in South Africa. *Lancet* 2021; **397:** 816–27.

# Effectiveness and cost-effectiveness of reactive targeted indoor residual spraying for malaria control in low transmission settings: Results from a cluster-randomised non-inferiority trial in South Africa

## Supplementary materials

**Contents**

S1: Template for Intervention Description and Replication (TIDieR) checklist .... 2
S2: Model parameters and assumptions .... 6
S3: Additional information on costing methods .... 9
S4: Decision tree .... 14
S5: Additional information on the calculation of disability-adjusted life-years .... 15
S6: Summary of trial protocol changes .... 16
S7: Trial profile .... 17
S8: Number of passively reported locally-acquired (blue) and imported (pink) malaria cases across both trial arms by month .... 18
S9: Secondary outcomes from endline survey .... 19
S10: Sensitivity analysis for primary outcome (malaria incidence) .... 20
S11: Rate difference between targeted IRS (TIRS) and standard IRS (SIRS) by (a) year and (b) province .... 21
S12: Total costs of standard IRS (SIRS) and targeted IRS (TIRS) strategies by cost component .... 22
S13: Cost-effectiveness acceptability curves .... 23
S14: Net cost savings from switching from standard IRS (SIRS) to targeted IRS (TIRS) .... 24
S15: Restricted cost-effectiveness analysis – Mpumalanga only .... 25
References .... 27

## S1: Template for Intervention Description and Replication (TIDieR) checklist

Source: Hoffmann et al. 2014.[1]

| **Item number / Name /** ***Description*** | **Intervention** | |
|---|---|---|
| | **Standard indoor residual spraying (SIRS)** | **Targeted indoor residual spraying (TIRS)** |
| 1 **Brief name:** *Provide the name or a phrase that describes the intervention* | Standard indoor residual spraying (SIRS) – **the control arm** | Targeted indoor residual spraying (TIRS) —**the intervention arm** |
| 2 **Why:** *Describe any rationale, theory, or goal of the elements essential to the intervention* | SIRS was standard practice in the provinces of Mpumalanga and Limpopo in South Africa before and during this study. Spraying insecticide on the interior walls of homes in malaria-risk areas is intended to kill female malaria vector mosquitoes that rest on walls either before or after taking a blood meal from inhabitants, and thereby reduce the risk of residents contracting or transmitting malaria. SIRS is deployed preventatively, commencing before the start of the annual transmission season to maximise impact and reduce transmission. As constrained budgets do not allow all homes to be sprayed, the ~30% of households deemed to be at greatest risk are informally targeted by the malaria control programme (MCP) managers each year. | Implementing untargeted IRS in areas where there is little evidence of recent transmission may be unwarranted and may result in sub-optimal quality of application of insecticide, and therefore effectiveness. Whilst spraying all houses in malarious areas would ensure maximum numbers of the population are living in insecticide-treated houses, this approach may be difficult to sustain against a background of constrained MCP budgets and substantially more expensive so-called 'next generation' insecticides. A reactive, targeted approach may therefore concentrate IRS efforts on those areas most in need, potentially leading to higher coverage and quality of spraying – and thus protection – amongst those most at risk, leading to comparable overall effectiveness relative to a more diffuse strategy. The substantial reduction in resources used for spraying would free up resources to be used more productively for other aspects of malaria control or in the wider health system. |
| 3 **What:** *Materials: Describe any physical or informational materials used in the intervention, including those provided to participants or used in intervention delivery or in training of intervention providers. Provide information on where the materials can be accessed (such as online appendix, URL)* | Each contract sprayer and assistant case investigator was provided with standard personal protective equipment, including a 2 conti suits (a one-piece protective garment with full-length sleeves and legs), jacket, cap, gloves, visor, boots, and masks. Case investigation team leaders received 1 conti suit.<br><br>Each contract sprayer was provided a 10 litre spray pump, sling bag, and galvanised bucket. We assumed one spray sheet provided per 3 contract sprayers.<br><br>We assumed that spray equipment used by case investigators would be taken from the stock provided to contract sprayers.<br><br>Environmental health practitioners and case investigation team leaders were each provided a Ford Ranger doublecab vehicle. Two Isuzu trucks were also used for mass spray operations, to transport sprayers and equipment.<br><br>SIRS used two types of insecticide: DDT and a pyrethroid (deltamethrin or alpha-cypermethrin). | Assistant case investigators and team leaders were provided with the same personal protective equipment, and assistant case investigators with the same spray equipment, as similar personnel in the SIRS arm.<br><br>Each team leader was provided a vehicle as in the SIRS arm.<br><br>Targeted spraying used deltamethrin only.<br><br>Training materials were developed by the trial team. |

| Item number / Name / *Description* | Intervention | |
|---|---|---|
| | **Standard indoor residual spraying (SIRS)** | **Targeted indoor residual spraying (TIRS)** |
| 4 **What:** *Procedures: Describe each of the procedures, activities, and/or processes used in the intervention, including any enabling or support activities* | Annual mass spray campaigns were conducted from August to December by the provincial MCPs. Spraying was conducted consistent with national guidelines. The number of 'structures' (broadly defined as individual rooms within a house) deemed eligible for spraying each year depended on the number of malaria cases in the previous season, MCP expert opinion, and available budget. Approximately one-third of households within the three malarious provinces were sprayed through an informal targeting process.<br><br>Case investigations were also conducted. Health workers who diagnosed a malaria case recorded relevant information into registers and reported to the relevant provincial MCP within 24 hours, which generated a report for case investigators to follow up and investigate. Unlike in usual practice, during the study, the provincial MCP contacted the study co-ordinator to determine whether cases were in the study area and, if so, in which arm. Case investigation teams comprised a team leader and assistant case investigators; in the SIRS arm, they were employed by the provincial MCP. They sought to determine if cases were locally acquired or imported and sprayed the index case household if it had not already been sprayed that season.<br><br>Malaria is notifiable by legal statute in South Africa. In malaria risk areas, clinical guidelines require all febrile patients presenting at health facilities be tested for malaria by rapid diagnostic tests (RDTs) (First Response™ Malaria Ag P. falciparum HRP2 Test, Premier Medical Corporation Ltd, India) or microscopy. Uncomplicated malaria cases are treated with artemether-lumefantrine (Coartem; Novartis Pharma AG). In 2019, single low dose primaquine, in addition to artemisinin-based combination treatment, was introduced in South Africa to reduce onward transmission of malaria,[2] however this was not available during the trial period. Intravenous artesunate is used to treat severe malaria. | No annual mass spray campaigns were conducted. Case investigations were triggered in the same way as in SIRS, but their conduct differed. In addition to usual case investigation activities, for local cases only, the teams sprayed the interiors of index case houses and up to 8 neighbouring houses (approximately 50 structures) within 200m with deltamethrin (regardless of wall type), subject to consent of householders.<br><br>Spraying was conducted consistent with national guidelines.<br><br>Anyone reporting fever in index and neighbouring households was tested and referred as in SIRS. |
| 5 **Who provided:** *For each category of intervention provider (such as psychologist, nursing assistant), describe their expertise, background, and any specific training given* | Annual mass spray campaigns were provided by teams of contract sprayers, employed for the season (110 days per year) by the provincial MCP. Supervision was provided by environmental health practitioners.<br><br>Case investigations were typically conducted in teams of 4, including 1 team leader and 3 assistant case investigators.<br><br>Spray personnel – primarily contract sprayers – attended 10 days of practical training on spraying methods, provided by the relevant provincial MCP. Training was delivered by environmental health practitioners. | Case investigation teams in TIRS clusters were employed by the trial.<br><br>Targeted spraying was provided in teams of 4, consisting of 1 team leader and 3 assistant case investigators.<br><br>Spray personnel – team leaders and assistant case investigators –attended 10 days practical training on spraying methods, provided by the relevant provincial MCP. Training was delivered by environmental health practitioners. |
| 6 **How:** *Describe the modes of delivery (such as face to face or by some other mechanism, such as internet or telephone) of the intervention and whether it was provided individually or in a group* | Spraying was conducted consistent with government standard practice: unpainted surfaces were sprayed with DDT at the start of the malaria season, and painted surfaces with a pyrethroid (deltamethrin or alpha-cypermethrin) after the start of the season.<br><br>Upon notification by a health facility of a local malaria case in a SIRS cluster, a case investigation team would attend and ask present household members to complete a short questionnaire, where they recorded demographic information and asked about recent travel movements. Febrile household members reporting current or recent fever were given a rapid diagnostic test and, if positive, treated according to national policy. | Case investigations were conducted as for SIRS, except that case investigation teams would spray the index case household and up to 8 neighbouring houses. Anyone reporting fever in index and neighbouring households were tested and referred as in SIRS. |

| **Item number / Name /** ***Description*** | **Intervention** | |
|---|---|---|
| | **Standard indoor residual spraying (SIRS)** | **Targeted indoor residual spraying (TIRS)** |
| 7 **Where:** *Describe the type(s) of location(s) where the intervention occurred, including any necessary infrastructure or relevant features* | The SIRS intervention took place in 31 clusters randomly selected out of a total of 62 clusters in Bushbuckridge and Phalaborwa sub-districts in Mpumalanga and Limpopo provinces, respectively, in north-eastern South Africa. The clusters were located in predominantly rural areas. A cluster was defined as groups of spray localities or complete wards to comprise populations of approximately 6,500 people. Eligible clusters included those with a reported incidence of malaria >1 and <5 cases per 1,000 per annum in at least one of the five years prior to the trial (2008-2012). | The TIRS intervention took place in the other 31 clusters. |
| 8 **When and how much:** *Describe the number of times the intervention was delivered and over what period of time including the number of sessions, their schedule, and their duration, intensity, or dose* | Mass spray campaigns were conducted as standard practice at the beginning of each of two malaria seasons: 2015/16 and 2016/17. Unpainted surfaces were sprayed with DDT at the start of the malaria season, and painted surfaces with a pyrethroid (deltamethrin or alpha-cypermethrin) after the start of the season.<br><br>Case investigations were conducted reactively throughout each malaria season, following notification of a local malaria case.<br><br>In SIRS clusters in Mpumulanga province, 128,519 and 90,196 structures were sprayed in years one and two, respectively. | Case investigations, including targeted spraying of index case and neighbouring households, were conducted reactively throughout the two malaria seasons, following notification of a local malaria case.<br><br>In TIRS clusters in Mpumalanga province, 132 and 6,163 structures were sprayed in years one and two, respectively. |
| 9 **Tailoring:** *If the intervention was planned to be personalised, titrated or adapted, then describe what, why, when, and how* | NA | NA |
| 10 **Modifications:** *If the intervention was modified during the course of the study, describe the changes (what, why, when, and how)* | In Mpumalanga province, the number of structures sprayed in SIRS clusters decreased from 128,519 in year one to 90,196 in year two. Spray data for SIRS clusters was not available for Limpopo province. | The trial protocol specified that, for the TIRS arm, a case investigation including targeted spraying would be triggered by two local cases, or one imported followed by one local case, within 0.5 km and four weeks of each other.<br><br>However, MCPs and local experts were concerned that this definition would not be sensitive enough and would result in local cases not receiving the intervention. As a result, during the pre-operations phase (prior to the commencement of the TIRS intervention), the team, in consultation with MCPs, the steering committee, the data safety monitoring committee, and local ethics committees decided to amend the trigger such that targeted spraying would be initiated for every local case reported. |

| **Item number / Name /** ***Description*** | **Intervention** | |
|---|---|---|
| | **Standard indoor residual spraying (SIRS)** | **Targeted indoor residual spraying (TIRS)** |
| 11 **How well:** *Planned: If intervention adherence or fidelity was assessed, describe how and by whom, and if any strategies were used to maintain or improve fidelity, describe them* | SIRS was intended to represent standard practice, and hence no additional quality checks were conducted. | In the TIRS arm, case investigations of local cases were designed to include spraying of the index case household plus 8 neighbouring households up to 200 metres from the index case household. Where occupants of households were not at home, case investigators were to make up to five call-backs, to ensure high coverage.<br><br>Using susceptible *Anopheles arabiensis* laboratory colony mosquitoes maintained at the provincial MCP insectary, standard cone bioassay testing took place within 2-4 weeks of spraying in a random sample of households in the TIRS arm to confirm the quality of IRS according to standard guidelines. Cone bioassay testing conducted on 14 sprayed structures in the TIRS arm reported an overall 24-hour mosquito mortality of 99.5% (N=420), providing assurance of satisfactory IRS application. |
| 12 **How well:** *Actual: If intervention adherence or fidelity was assessed, describe the extent to which the intervention was delivered as planned* | NA | An average of 3.7 neighbouring houses, including outbuildings, were sprayed per case investigation. Case investigators reported that neighbours were often not at home and there was not sufficient time for a return visit. In addition, some index cases were reported from fairly newly-built dwellings situated in areas with <8 sprayable neighbouring households with 200m radius. |

## S2: Model parameters and assumptions

All costs are presented in constant 2017 United States dollars. * Mean of cluster incidences. NA: not applicable. s.d.: standard deviation. SIRS: standard IRS. TIRS: targeted IRS. CFR: case fatality rate. DSA: deterministic sensitivity analysis. EHP: environmental health practitioner. MCP: Malaria Control Programme. RDT: rapid diagnostic test. SA: South Africa. WHO: World Health Organization.

| Parameter | Year(s) | Base case | Probabilistic | | Deterministic | | Source for base case (source for range) |
|---|---|---|---|---|---|---|---|
| | | | Distribution | s.d. | Low | High | |
| **General** | | | | | | | |
| Discount rate | Both | 3.0% | NA | NA | 1.0% | 10.0% | Wilkinson et al. 2016 [3] (standard assumptions) |
| Population – Ehlanzeni district | Both | 1,754,931 | NA | NA | NA | NA | Statistics SA demographic data [4]; (2016) |
| Population – Bushbuckridge sub-district | Both | 546,215 | NA | NA | NA | NA | Statistics SA demographic data [4]; (2016). Includes population outside study clusters |
| Population – SIRS trial clusters | Both | 189,150 | NA | NA | NA | NA | Trial data |
| Population – TIRS trial clusters | Both | 204,237 | NA | NA | NA | NA | Trial data |
| **Effects** | | | | | | | |
| Incidence rate – SIRS (local cases per 1000py)* | 1 | 0.063 | Gamma | 0.032 | 0.001 | 0.125 | Trial estimates based on passive surveillance (95% CI) |
| | 2 | 1.83 | Gamma | 0.37 | 1.10 | 2.56 | |
| | Both | 0.95 | Gamma | 0.19 | 0.58 | 1.32 | |
| Rate ratio (adjusted for province) | 1 | 1.81 | Normal | 0.97 | 0.72 | 4.53 | Trial estimates based on passive surveillance (95% CI) |
| | 2 | 1.21 | Normal | 0.09 | 1.04 | 1.41 | |
| | Both | 1.22 | Normal | 0.09 | 1.05 | 1.42 | |
| Proportion of cases that become severe | Both | 0.155 | Beta | 0.040 | 0.078 | 0.233 | Mpumalanga MCP case records (+/- 50%) |
| Proportion of all cases that result in death (case fatality rate) | Both | 0.030 | Beta | 0.006 | 0.0079 | 0.04 | Trial estimates based on passive surveillance (95% CI; DSA: SA malaria CFR (2016) [5], assumption) |
| Duration of uncomplicated malaria (days) | Both | 3.0 | Gamma | 0.4 | NA | NA | Assumption (+/- 25%) |
| Duration of severe malaria (days) | Both | 7.0 | Gamma | 0.9 | NA | NA | Assumption – inpatient stay + 2 days (+/- 25%) |
| Disability weight: uncomplicated malaria | Both | 0.051 | Beta | 0.011 | NA | NA | Salomon et al. 2015 [6], 'infectious disease moderate' |
| Disability weight: severe malaria | Both | 0.133 | Beta | 0.026 | NA | NA | Salomon et al. 2015 [6], 'infectious disease severe' |
| Years of life lost per death (discounted) | Both | 19.8 | Normal | 4.4 | NA | 24.2 | Trial data on sex & age at death; [7], South Africa life-expectancy (DSA: Japanese life-expectancy) |
| **Annual economic costs of interventions** | | | | | | | |
| ***SIRS*** | | | | | | | |
| Spray operations per 100,000 population | 1 | $123,252 | Gamma | $15,721 | NA | NA | Modelled from primary cost analysis (s.d.=25% base case / 1.96) |
| | 2 | $109,868 | Gamma | $14,014 | NA | NA | |
| | Both | $116,388 | Gamma | $14,846 | NA | NA | |
| Case investigations per 100,000 population | Both | $65,934 | Gamma | $8,410 | NA | NA | Modelled from primary cost analysis (s.d.=25% base case / 1.96) |
| ***TIRS*** | | | | | | | |
| Set-up per 100,000 population (annualised) | Both | $2,120 | Gamma | $270 | NA | NA | Modelled from primary cost analysis (s.d.=25% base case / 1.96) |
| Spray operations per 100,000 population | Both | $17,804 | Gamma | $2,271 | NA | NA | EHPs retained from SIRS arm. Modelled from primary cost analysis (s.d.=25% base case / 1.96) |

| Parameter | Year(s) | Base case | Probabilistic | | Deterministic | | Source for base case (source for range) |
|---|---|---|---|---|---|---|---|
| | | | Distribution | s.d. | Low | High | |
| Case investigations per 100,000 population | 1 | $65,339 | Gamma | $8,334 | NA | NA | Modelled from primary cost analysis (s.d.=25% base case / 1.96) |
| | 2 | $67,056 | Gamma | $8,553 | NA | NA | |
| | Both | $66,184 | Gamma | $8,442 | NA | NA | |
| **Key intervention cost components** | | | | | | | |
| Average annual salary: EHP | Both | $32,416 | NA | NA | NA | NA | Mpumalanga MCP personnel records |
| EHPs per 100,000 population retained in TIRS | Both | 0.55 | NA | NA | 0.41 | NA | Mpumalanga MCP personnel records (DSA: -25%) |
| Average annual salary: team leader | Both | $22,586 | NA | NA | NA | NA | Mpumalanga MCP personnel records |
| Average annual salary: assistant case investigator | Both | $12,079 | NA | NA | NA | NA | Mpumalanga MCP personnel records |
| Case investigators per 100,000 population required in TIRS | Both | 3.92 | NA | NA | 3.52 | 4.90 | Trial personnel records (DSA: -10%, +25%). Case investigators include team leaders and assistant case investigators |
| Additional costs of salaried employees (% salary cost) | Both | 43% | NA | NA | NA | NA | Mpumalanga MCP financial accounts; includes: social security contributions, performance bonus, housing allowance, service bonus, and other benefits |
| Daily rate: contract sprayer | Both | $20.15 | NA | NA | NA | NA | Mpumalanga MCP financial accounts |
| Contract sprayer-days per spray season | 1 | 13,750 | NA | NA | 10,313 | 17,188 | Mpumalanga MCP financial accounts (DSA: +/- 25%). Sprayer days = number of contract sprayers employed multiplied by number of days in the annual mass spraying season |
| | 2 | 14,300 | NA | NA | 10,725 | 17,875 | |
| | Both | 14,025 | NA | NA | 10,519 | 17,531 | |
| DDT cost per kg (SIRS arm only) | Both | $15.57 | NA | NA | $12.46 | $31.14 | Mpumalanga MCP financial accounts (DSA: assumptions; -20%, +100%. Cost per kg of DDT and deltamethrin are varied together in DSA (see Table 3)) |
| Structures sprayed per kg of DDT (SIRS arm only) | Both | 9.8 | NA | NA | NA | NA | Mpumalanga MCP spray records (kg used / structures sprayed) |
| Deltamethrin cost per kg | Both | $98.71 | NA | NA | $78.97 | $197.43 | Mpumalanga MCP financial accounts (DSA: assumptions; -20%, +100%. Cost per kg of DDT and deltamethrin are varied together in DSA (see Table 3)) |
| Structures sprayed per kg of deltamethrin | Both | 225.6 | NA | NA | NA | NA | Mpumalanga MCP spray records (kg used / structures sprayed) |
| SIRS structures sprayed with DDT (% total SIRS structures sprayed) | 1 | 17% | NA | NA | NA | NA | Mpumalanga MCP spray records, Bushbuckridge |
| | 2 | 13% | NA | NA | NA | NA | |
| | Both | 15% | NA | NA | NA | NA | |
| **Diagnostic and treatment costs** | | | | | | | |
| Cost per uncomplicated case | Both | $8.74 | Gamma | $1.12 | NA | NA | Sum of constituent costs: outpatient visit, RDT, artemether-lumefantrine (s.d.=25% base case / 1.96) |
| Cost per severe case | Both | $64.18 | Gamma | $8.19 | NA | NA | Sum of constituent costs: inpatient stay, RDT, artesunate, artemether-lumefantrine (s.d.=25% base case / 1.96) |
| **Diagnostic and treatment cost components** | | | | | | | |

| Parameter | Year(s) | Base case | Probabilistic | | Deterministic | | Source for base case (source for range) |
|---|---|---|---|---|---|---|---|
| | | | Distribution | s.d. | Low | High | |
| Cost of RDT (antigen Pf – point-of-care test, 25 x 1 test) | Both | $0.35 | NA | NA | NA | NA | Global Fund reference pricing [8]; +10% shipping cost |
| Cost of artemether-lumefantrine (20/120mg, 6x4, non-dispersible tablets) | Both | $0.63 | NA | NA | NA | NA | Global Fund reference pricing [9]; assume all adult treatment; 1 blister pack per treatment; +10% shipping cost |
| Cost of outpatient visit | Both | $8.17 | NA | NA | NA | NA | WHO health service costs [10]; South Africa, health centre no beds |
| Cost of inpatient bed-day | Both | $60.96 | NA | NA | NA | NA | WHO health service costs[10]; South Africa, secondary level hospital |
| Cost of artesunate (120mg powder for solution for injection)(/120mg, 6x4, non-dispersible tablets) | Both | $7.98 | NA | NA | NA | NA | Global Fund reference pricing [9]; assume all adult treatment; 3 vials per treatment; +10% shipping cost |
| Length of inpatient stay (days) | Both | 5 | NA | NA | NA | NA | Assumption |

**S3: Additional information on costing methods**

## 1. Implementation costs

For both standard and targeted indoor residual spraying (IRS) strategies (SIRS and TIRS, respectively), we estimated the cost of implementation for a standardised population of 100,000 to ensure that any differences in costs between the arms reflected differences driven by the choice of strategy rather than differences in the population of the clusters in which the strategies were implemented. We assumed that our standardised 100,00 population comprised the population of a single sub-district nested within a single province. Costs were estimated based on data for Mpumalanga province only (where 49 of the 62 clusters were located) because detailed accounts from the Limpopo MCP were not available.

For both strategies, we also estimated costs of implementation by the provincial malaria control programme (MCP) to ensure that we usefully informed the decision facing the MCP. As the MCP implemented SIRS during the trial, no adjustment was required; however, as the trial team implemented TIRS, we adjusted TIRS costs to best approximate 'real world' implementation as part of the provincial malaria control programme (MCP). All resources in both arms were costed using unit costs from the Mpumalanga MCP, even where the trial obtained them (for TIRS) at a different unit cost. We assumed that implementation of TIRS would not change MCP management and overheads.

The following cost components were identified and described in greater detail below:

- personnel (including permanent employees and contract sprayers);
- insecticide;
- equipment; and
- transport.

### *1.1. Personnel*

Employees and contractors involved in delivering the interventions (SIRS or TIRS) were identified in consultation with Mpumalanga MCP and trial personnel.

For the SIRS arm, employees were identified at the Bushbuckridge sub-district. Mass spray operations over the trial period were conducted by contract sprayers (125 in year one, 130 in year two) engaged for 110 days (including training) each year. Contract sprayers in Bushbuckridge were overseen by 3 environmental health practitioners (EHPs) employed by the MCP. Although EHPs perform functions other than spray operations (e.g. disease surveillance, case investigations) – particularly outside the spray season – such allocation is difficult to estimate without a detailed time-and-motion study, which was not conducted as part of the trial. We therefore assumed EHPs were wholly engaged in managing the spray operations programme. This assumption may therefore have led us to over-estimate the costs of SIRS; however, as we also assumed the EHPs would be employed under the TIRS strategy (see below), this assumption did not affect estimates of the difference in costs between SIRS and TIRS. Case investigations in Bushbuckridge over the trial period were conducted by 16 assistant case investigators and 6 team leaders (together referred to as "case investigators" or "case investigation teams"). Like EHPs, case investigation teams also conducted activities not directly related to case investigations (e.g. larviciding); however,

for the purposes of the analysis, we assumed these personnel were wholly engaged in case investigation activities. As a result, the cost of case investigations may be overstated for SIRS; however, the same assumption was made for case investigation personnel in the TIRS arm.

For the TIRS arm, two case investigation teams were employed by the trial to investigate cases in TIRS clusters in the Bushbuckridge sub-district of Mpumalanga Province. (An additional team, which conducted case investigations in Limpopo Province, was not included in the costing as costs and other data relating to SIRS clusters in Limpopo were not available.) Each team comprised 1 team leader and 3 assistant case investigators. In addition, we also conservatively assumed (in our base case analysis) that EHPs overseeing the SIRS spray operations programme would continue to be employed if TIRS were implemented by the MCP. This conservative assumption may overestimate the costs of TIRS and thus underestimate the potential resource savings in switching from SIRS to TIRS. We assumed that switching from SIRS to TIRS would not change MCP management and overhead resources at either the sub-district or province level. We considered this assumption reasonable and conservative, particularly as the adoption of TIRS would be unlikely to substantially change the number of employees managed by the MCP (except for contract sprayers, who would no longer be employed).

To estimate the number of personnel required to deliver either SIRS or TIRS in a standardised population of 100,000, we calculated the number of each personnel group per head of population. For SIRS, the number of Mpumalanga MCP personnel working in Bushbuckridge sub-district (EHPs, contract sprayers, SIRS team leaders, SIRS assistant case investigators) were divided by the estimated Bushbuckridge population (546,215 in 2016)[4]. We used the total Bushbuckridge population for this calculation, even though Bushbuckridge included both SIRS and TIRS clusters (and other areas outside the trial clusters); we considered this appropriate as the number of Bushbuckridge personnel did not materially change either before or after the trial. If anything, this assumption may therefore underestimate the costs of SIRS per head of population during the trial period, and is therefore conservative. For TIRS, the number of personnel employed by the trial in the TIRS arm (team leaders and assistant case investigators) were divided by the population in the TIRS clusters (204,237). We also assumed the same number of EHPs in TIRS as calculated for SIRS, as we had assumed that EHPs would be retained if TIRS were implemented by the MCP.

All personnel in both TIRS and SIRS arms were costed at Mpumalanga MCP pay rates, even though TIRS spray teams were employed by the trial. Employment costs were calculated inclusive of additional costs of employment (including social security contributions, performance bonus, housing allowance, service bonus, and other benefits), which we calculated for all permanent employees (i.e. excluding contract sprayers) as a percentage of direct salaries.

### *1.2. Insecticide*

In the SIRS arm, structures (broadly defined as individual rooms within a house) were sprayed as part of a mass spraying campaign at the start of each malaria season. Unpainted structures were sprayed with DDT (17% of structures sprayed in year one, 13% in year two) and painted surfaces (in Mpumalanga Province) with deltamethrin. From Mpumalanga MCP spray records, which provide data on the number of structures sprayed and kilograms of insecticide used, we calculated the average number of structures sprayed per kilogram of DDT (9.8 structures) and deltamethrin (225.6 structures) in Bushbuckridge sub-district across the two-year trial period. We

estimated the amount of each insecticide used for the standardised 100,000 population by multiplying the average number of structures sprayed per kilogram (calculated above) by the number of structures sprayed (assuming the proportions of structures sprayed with each type of insecticide were the same as in the trial).

In the TIRS arm, structures were sprayed as part of targeted spraying conducted during case investigations, using deltamethrin only. We assumed the average number of structures sprayed per kilogram of deltamethrin was the same as in the SIRS arm. Total insecticide used in the TIRS arm was estimated as the average number of structures sprayed per kilogram of deltamethrin multiplied by the number of structures sprayed in the TIRS arm for the standardised 100,000 population.

For both arms (DDT and deltamethrin for SIRS, only deltamethrin for TIRS), the cost of insecticide per kilogram was calculated from Mpumalanga MCP financial records and discussions with finance personnel as the average cost per kilogram paid by the MCP across the two-year study period.

### *1.3. Equipment*

From discussions with the manager of the Mpumalanga MCP, we identified spray equipment and personal protective equipment (PPE) required by SIRS spray personnel, and the unit cost and estimated useful life of each item. The cost of each item was annualised based on its useful life (10 years for spray pumps and 3 years for other spray equipment – spray sheets, sling bags, galvanised buckets). All other items (including conti suits (one-piece protective garments with full-length sleeves and legs), boots, gloves, visors, and masks) were assumed to be replaced each season or (for visors and masks) more frequently.

For the SIRS arm, we assumed that all contract sprayers required spray equipment and PPE. Although we assumed that SIRS case investigation teams did not conduct targeted spraying, we conservatively assumed that assistant case investigators received the same PPE as contract sprayers; however, we assumed any spray equipment required for targeted spraying was taken from the equipment used by the mass spray programme. We assumed that team leaders received 2 conti suits per season but no other PPE.

For the TIRS arm, we assumed that team leaders and assistant case investigators used the same PPE as in the SIRS arm, and that assistant case investigators used the same spray equipment as for contract sprayers.

For each arm, the annualised cost of spray equipment and PPE was calculated as the number of relevant personnel of each type (team leaders and assistant case investigators and, for the SIRS arm, contract sprayers) as calculated above for the standardised 100,000 population, multiplied by the number of each item of equipment required annually by each person, multiplied by the annualised cost of each item of equipment.

### *1.4. Transport*

Transport comprises two different types of costs:

- capital cost of purchasing vehicles; and
- vehicle recurrent costs (fuel, maintenance, and fleet management).

In the SIRS arm, each of the 3 EHPs and 4 case investigation team leaders were allocated a Ford Ranger double-cab, and 2 Isuzu trucks were also used for spray operations. We calculated the annualised cost of these vehicles,

based on the purchase date and purchase price, and assuming a useful life of 10 years. Similar to the assumptions made for personnel, we assumed that the vehicles were wholly used for either mass spraying or case investigation activities.

In the TIRS arm, each of the 2 case investigation teams in Bushbuckridge used a utility vehicle, which was purchased for the purposes of the trial. However, in order to model the 'real-world' scenario in which the provincial MCP switched from SIRS to TIRS, we assumed the annualised cost of each vehicle was the same as calculated for the SIRS arm.

For each arm, the annualised cost of vehicles was calculated as the number of vehicles required by relevant personnel of each type (EHPs and case investigation team leaders) as calculated above for the standardised 100,000 population, multiplied by the annualised cost of each vehicle.

Vehicle logs were not available for vehicles in either the SIRS or the TIRS arm. Total vehicle recurrent costs (fuel, maintenance, and fleet management) were obtained for the Mpumalanga MCP.

The annual recurrent cost of vehicles was identified at the province level from Mpumalanga MCP financial records, calculated as the average of the two years of the trial period. Costs could not be apportioned to vehicles in the Bushbuckridge sub-district used for relevant activities in the trial (spray operations and case investigation) on a per kilometre basis, because log books were not available for individual vehicles. Instead, a share of the Mpumalanga province's annual vehicle recurrent costs was allocated to the Bushbuckridge sub-district based on population; specifically, as a proportion of the population in Bushbuckridge (546,215 in 2016)[4] to the population in the Ehlanzeni district (1,754,931 in 2016, which includes the three sub-districts with the highest malaria transmission in the province and the focus of the Mpumalanga MCP's activities).[4] We divided the recurrent annual vehicle costs allocated to Bushbuckridge sub-district by the total number of (government-owned and employee-owned) vehicles used for MCP operations in Bushbuckridge (11) to obtain an annual recurrent cost per vehicle. This annual recurrent cost per vehicle was then multiplied by the vehicles allocated to activities included in the trial for each arm: for SIRS, the vehicles used for spray operations or case investigations in Bushbuckridge, and for TIRS, the vehicles used for case investigations in the TIRS clusters. These costs were then adjusted by population (for SIRS, the Bushbuckridge population; for TIRS, the population in the TIRS clusters), to estimate the cost for the standardised 100,000 population.

### *1.5. Set-up*

We assumed that there were no set-up costs associated with SIRS, because it is a long-running programme and therefore would not need an up-front investment to continue. For TIRS, we estimated the costs that would be associated with setting up TIRS under the management of the provincial MCP, including time attending meetings, planning, delivering and receiving training, as well as the development of materials. All set-up costs were annualised over three years (that is, assuming that similar such activities would need to be conducted on a 3-yearly basis).

We assumed that set-up costs would be incurred at the province, district, and sub-district levels. At the province level, we assumed 20 hours for the MCP deputy director and each of the senior management – 2 assistant directors, 2 control EHPs (senior EHPs who manage individual districts), and 1 senior administration officer – to attend

province-level meetings and review materials. At the district level, we assumed 20 hours for the control EHP in charge of the district to attend sub-district level meetings.

We estimated sub-district set-up costs based on the number of EHPs, team leaders, and assistant case investigators in Bushbuckridge sub-district (3, 6, and 16, respectively), and then scaled these costs to the standardised 100,000 population. We assumed 20 hours for each of the 3 EHPs to attend sub-district-level meetings and plan implementation, and an additional 40 hours each for 2 EHPs to deliver training. For each case investigation team member (team leaders and assistant case investigators), we assumed 10 hours to attend sub-district-level meetings, plus 40 hours of training. These costs were converted to costs per head of population, and then multiplied by 100,000 to obtain costs for the standardised population.

We also included ZAR10,000 (2016) (US$790, constant 2017) per sub-district for development of materials.

## 2. Inflation and foreign exchange

We converted all costs to constant 2017 United States dollars (USD) to account for variation in inflation and exchange rates over time. As recommended,[11] we performed this procedure separately depending upon whether the resource was internationally traded or not.

For internationally traded resources (assumed to include insecticide, vehicle capital, diagnostic tests, and drugs), we first converted all costs to US dollars using the average official foreign exchange rate for the year in which the costs were incurred, and then inflated costs to 2017 terms using US GDP deflators.[12,13]

For resources not internationally traded (assumed to include salary costs of all personnel , all spray equipment and PPE costs, fuel and maintenance costs for vehicles, and health service outpatient consultation and inpatient bed-day costs), we first inflated costs to 2017 terms using South African GDP deflators, and then converted costs to US dollars using the average official foreign exchange rate for 2017.[12,13]

The average official exchange rate in 2017 was 13.324 South African rand for 1 US dollar.[13]

**S4: Decision tree**

The decision tree model structure shows the decision node (square), representing the policy choice of whether to implement standard IRS (SIRS) or targeted IRS (TIRS), followed by a series of chance nodes (circles) describing the pathway to final health outcomes (no malaria, recovery, or death). The model assumes that all cases are passively detected and receive malaria treatment, with uncomplicated malaria cases treated as outpatients and severe cases as inpatients.

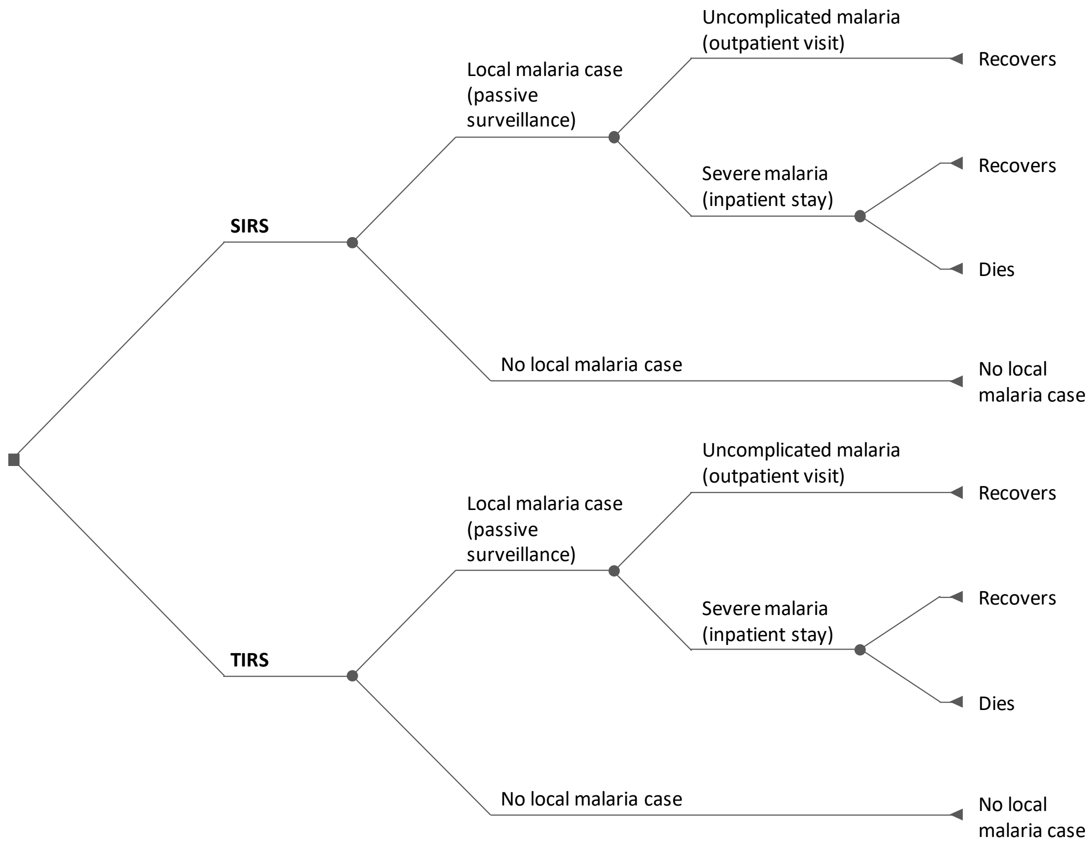

**S5: Additional information on the calculation of disability-adjusted life-years**

Disability-adjusted life-years (DALYs) were modelled as the sum of years of life lost (YLLs) and years of life with disability (YLDs) using a discount rate of 3% and no age weighting.[3]

YLDs were estimated as the product of the number of YLDs per case and the number of cases under each strategy. YLDs per case of uncomplicated or severe malaria were calculated as the product of the estimated duration of illness and relevant disability weights. Cases were estimated for each arm in each study year and for the two-year study period. Cases associated with SIRS were estimated as the crude incidence of local cases across both provinces (Table 1) scaled up to the standardised 100,000 population. Cases associated with TIRS were estimated as the product of the number of cases estimated for the SIRS arm and the adjusted rate ratio for both provinces combined.

YLLs were estimated as the product of the number of YLLs per death and the number of deaths under each strategy. YLLs per death were calculated as the mean discounted remaining life-expectancy for each of the deaths recorded in the study across both arms, based on age at death and South African life tables.[7] The case fatality rate was assumed to be the same in both arms and both years, calculated as total recorded deaths from locally-acquired malaria divided by total passively detected local cases (i.e., the same case definition used in calculating case incidence).

**S6: Summary of trial protocol changes**

An amendment application detailing the following minor changes to the trial protocol was submitted to the LSHTM Observational / Interventions Research Ethics Committee on 7 October 2015. Ethics approval for the amendment was provided on 10 December 2015.

---

1. In consultation with the provincial malaria control managers the **study area** has been re-drawn to include only the municipalities of Phalaborwa and Bushbuckridge. Areas further north in Limpopo province were felt to be unsuitable due to the risk of higher malaria incidence in some years (>5 per 1000 pa) and the Nkomaz area of Mpumalanga was unsuitable since most malaria cases in this area are imported cases (see map Figure 4.2.1 on page 13 of the original protocol).

2. The **trial schedule** has been changed with randomisation delayed by 12 months. This change has been approved by the trial steering committee by e-mail correspondence with all independent members of the TSC. The reason for the delay was to assemble better information on malaria incidence in the study area, to form clusters with appropriate population size and a history of low (but not zero) malaria incidence, and to conduct a baseline survey in these study clusters. The **number of study clusters** has been increased to 62 (31 clusters per arm).

3. **Constrained randomisation** of the clusters to the two study arms has been carried as specified in the protocol, page 14. The variables that have been used to balance the study arms were: malaria incidence, province, cluster population density, cluster size and total length of rivers and streams in the cluster. This is different from the proposed list given on page 14 of the original protocol which stated that malaria incidence, province, proportion of imported cases, elevation, proximity to international borders and urban/rural would be used. The variables in the original list (apart from malaria incidence and province) were not used because it was considered that they did not vary substantially, and because the new list was considered more relevant to the trial outcomes.

4. Details regarding **case investigation and reactive spraying** have been laid out in step-by-step detail, in consultation with provincial malaria control managers. The precise procedure is attached, and has been incorporated into the amended protocol as appendix 1. In brief, reactive spraying will be carried out whenever a local case arises in the targeted IRS arm within 4 weeks and within 0.5km of another case as laid out in the original protocol; however, if there is any doubt about whether the previous case meets this criterion, then reactive IRS will be carried out anyway. Whilst the original protocol stated that spraying will be carried out within a radius of 0.5km of the index case, the revised procedure provides for spraying of 50 structures around the index case, up to 200 from the index case household. However, if additional cases are encountered in the neighbourhood, a similar process will be followed in the neighbourhood of this case. Neighbourhood investigations will be carried out in eight neighbouring households (as opposed to 30 specified in the original protocol). This revision was made to make the method of reactive spraying sustainable, and because research in Swaziland and Namibia (pers. comm. unpublished data, Davis Mumbengegwi) has shown that secondary cases are almost always within the index case household, or immediately surrounding houses. The case investigation and targeted IRS will be assisted by a tablet computer app that is being developed for the purpose.

5. Details of **entomological monitoring** have been re-formulated in detail and now replace the original appendix 5.

6. The original protocol refers to a **previous outbreak of cases** in Bushbuckridge and Phalaborwa in early 2014. A report regarding this outbreak is attached, and forms appendix 9 of the amended protocol.

7. Minor modification has been made to the **DSMC charter**, at the request of the latter (appendix 7).

## S7: Trial profile

SIRS: standard indoor residual spraying. TIRS: targeted indoor residual spraying. N/A: Data not available.

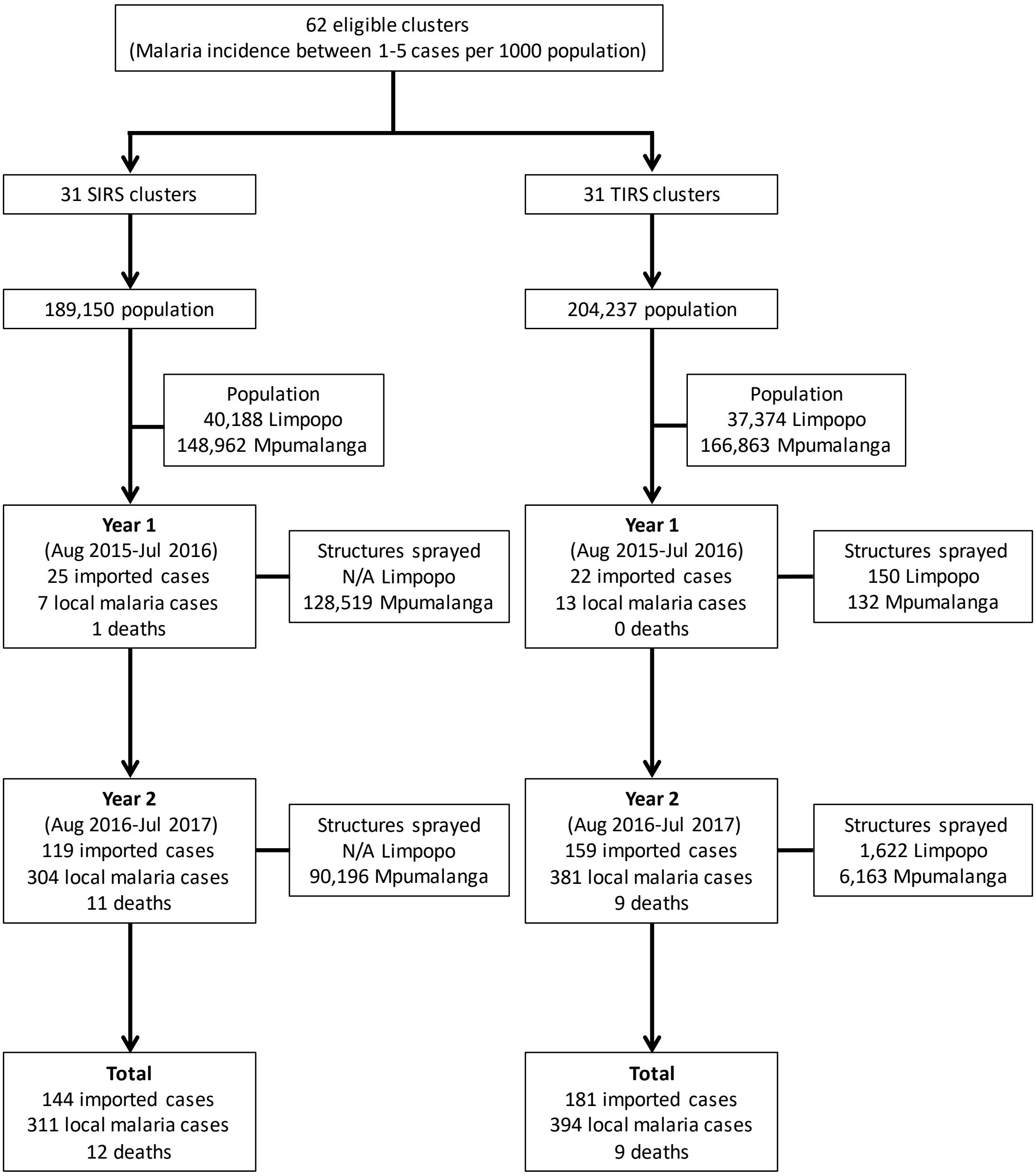

**S8: Number of passively reported locally-acquired (blue) and imported (pink) malaria cases across both trial arms by month**

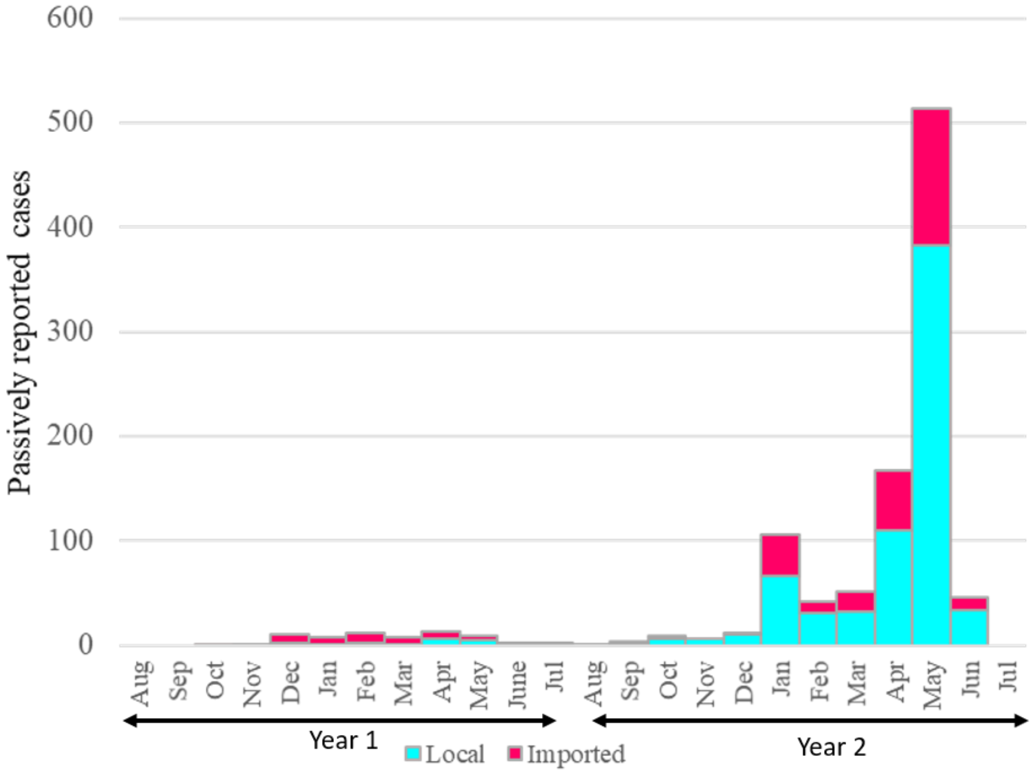

**S9: Secondary outcomes from endline survey**

In a cross-sectional household survey conducted between April and July 2017, study teams visited 1,290 randomly selected households in the study area, across all 62 trial clusters (31 and 31 in standard indoor residual spraying (SIRS) and targeted indoor residual spraying (TIRS) arms, respectively). Sampling was based on detailed maps of housing structures in the area. Of the selected houses, 420 were empty, abandoned, were not residential buildings, did not have adults present, or refused to take part. Of the 870 houses remaining, 56% (n=490) and 44% (n=380) were in SIRS and TIRS clusters, respectively. After seeking written informed consent from the head of household, or their representative, a structured questionnaire was administered seeking information on knowledge, attitudes and practices related to indoor residual spraying (IRS).

Householders were asked whether their house had been sprayed in the previous 12 months. In the SIRS arm 30% (n=141) reported that their houses had been sprayed, compared to 5% (n=20) in the TIRS arm. Of those that had been sprayed, 89% (n=122) and 85% (n=17) reported being satisfied with the spraying in the SIRS and TIRS arms, respectively. Of those that had been sprayed, 19% reported that they had replastered or washed their walls since the spraying (16% (n=22) and 40% (n=8) in SIRS and TIRS, respectively), in contravention of guidance.

96% (n=469) and 88% (335) of householders in the SIRS and TIRS arms respectively said they would like their house to be sprayed in the next round. The main reason that householders gave was because they felt that there were still mosquitoes (or other insects) in the house (69% (n=338) and 76% (n=289) in SIRS and TIRS, respectively).

The majority of householders had a preference for mass annual spray campaigns (44% (n=216) and 49% (n=185) in SIRS and TIRS, respectively) over targeted spraying (19% (n=95) and 22% (n=83) in SIRS and TIRS, respectively). For those that preferred mass spraying, the majority liked it because it kills other insects (33%) or protects people from mosquitoes (31%) (similar results by arm).

Filter paper blood spots were taken from all consenting residents within households to investigate serological responses to *P. falciparum* antigens. Serological responses to AMA-1 and MSP-119 were measured using enzyme-linked immunosorbent assay (ELISA). No molecular testing for parasites was undertaken due to the presumed extremely low prevalence of infections. A total of 1,752 samples were collected from 772 households. Seropositivity to both antigens was low (5% to AMA-1 and 9% to MSP-119) with no children under 5 recorded as positive to either antigen. Seropositivity to either antigen was slightly higher in the SIRS arm (11.2%) than the TIRS arm (8.9%). Seropositivity increased with age, reflecting lifetime exposure to malaria.

**Conclusions**

IRS was generally viewed favourably by householders. The majority were keen to have their house sprayed in the next round, for reasons not always related to malaria prevention. The method of spraying they received during the trial did not appear to impact on householders' opinions of IRS. One in five householders were not compliant with the IRS teams' instructions to not wash or replaster the walls after spraying; this was more prevalent in the TIRS arm (though numbers were small). Further serological analysis will be undertaken and reported in separate publications.

**S10: Sensitivity analysis for primary outcome (malaria incidence)**

We additionally performed the main analysis adjusting for all factors included in the restricted randomisation (mean malaria incidence between 2010-15, province, population size, proportion of households sprayed with IRS in 2014, population density, and the total length of streams and rivers). The adjusted analysis did not change the overall outcome, resulting in a rate difference of 0.092 (-0.313-0.497), $p<0.001$ compared to margin of non-inferiority, indicating that targeted IRS (TIRS) was significantly non-inferior to standard IRS (SIRS).

**S11: Rate difference between targeted IRS (TIRS) and standard IRS (SIRS) by (a) year and (b) province**

Large vertical bars represent 95% confidence intervals (CIs) and smaller vertical bars represent 90% CIs. Dashed red vertical lines represent the non-inferiority margin (1/1,000 population increase in incidence). In non-inferiority tests, the two-sided 95% CIs presented correspond to one-sided 97.5% CIs, and the two-sided 90% CIs presented correspond to one-sided 95% CIs. Note: the trial was not powered to demonstrate non-inferiority in individual years, nor in each province.

**a) Rate differences by year, crude and adjusted by province**

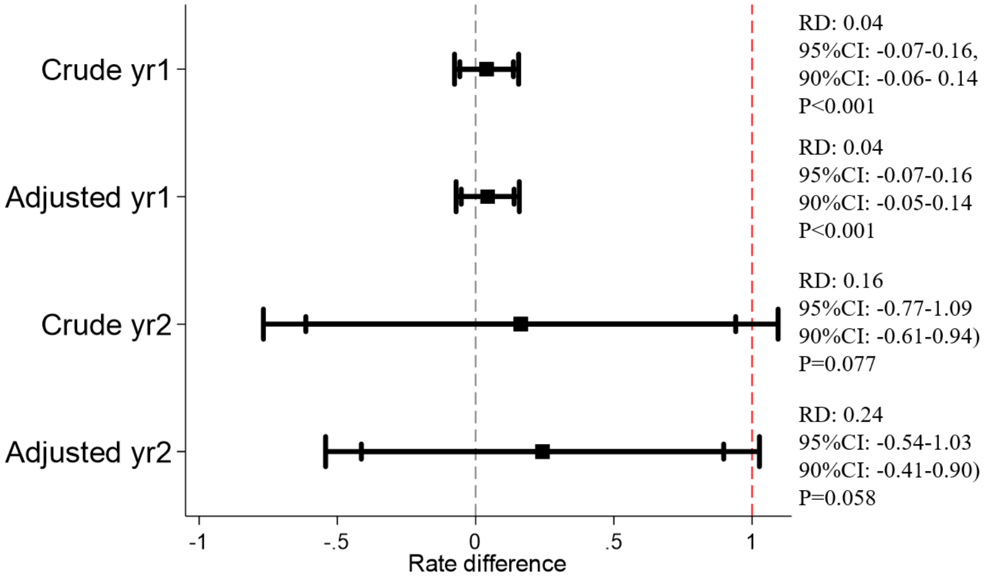


**b) Rate differences by province**

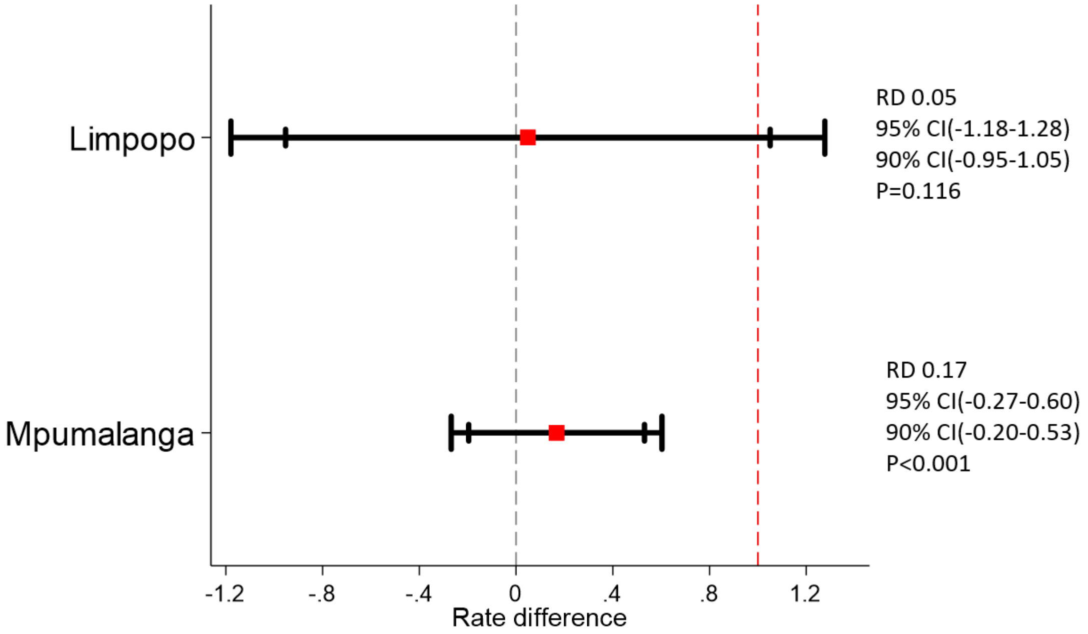

## S12: Total costs of standard IRS (SIRS) and targeted IRS (TIRS) strategies by cost component

The figures show the total annual economic cost under each of the strategies (SIRS and TIRS), broken down by cost component, in (a) year one, (b) year two, and (c) the two-year study period. They show that the TIRS strategy incurred substantially lower costs than SIRS in both years primarily because TIRS did not involve hiring contract sprayers and used far less insecticide.

### (a) Total costs in year 1

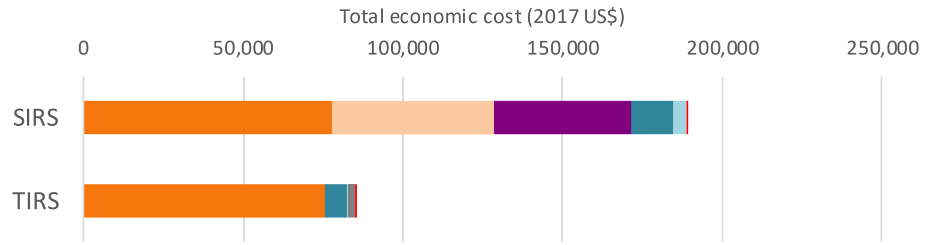


### (b) Total costs in year 2

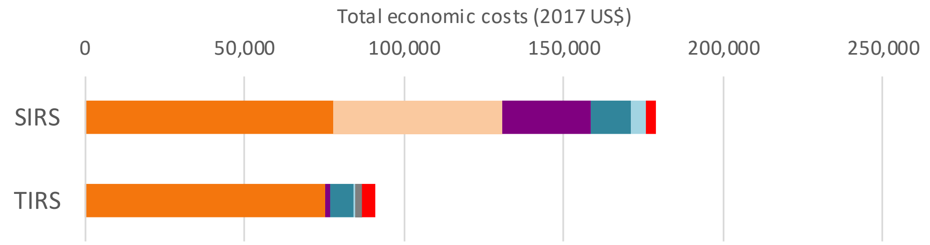


### (c) Annual costs across the two-year study

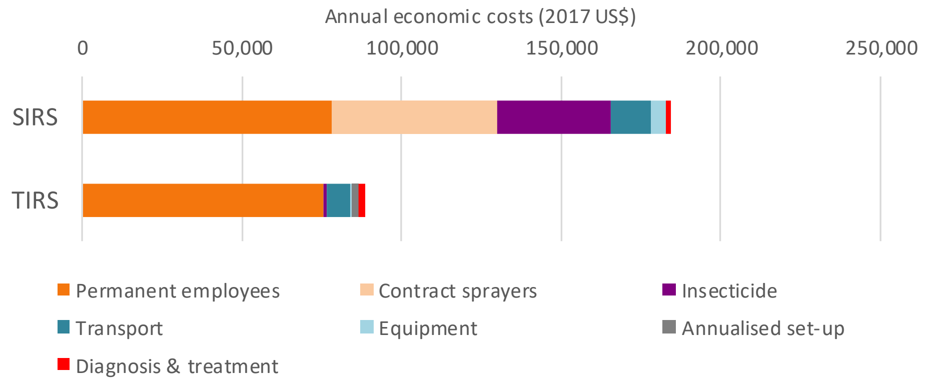

## S13: Cost-effectiveness acceptability curves

The figure shows the probability that switching from standard IRS (SIRS) to targeted IRS (TIRS) is cost-effective, at different cost-effectiveness thresholds, for years one and two individually and both years combined.

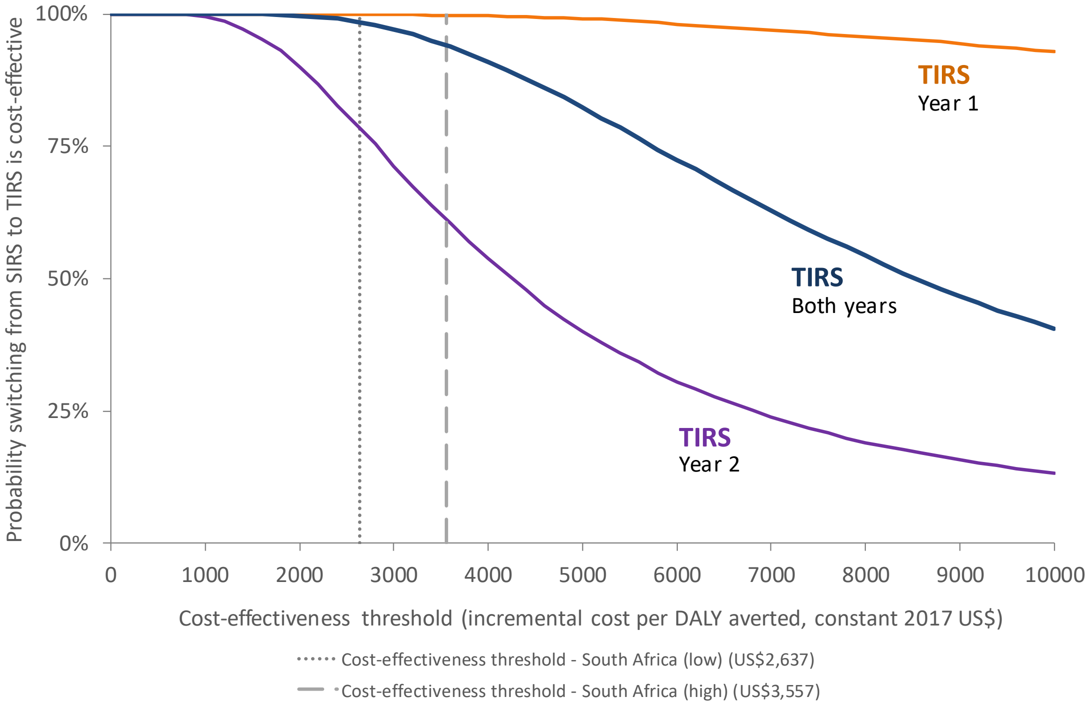

### S14: Net cost savings from switching from standard IRS (SIRS) to targeted IRS (TIRS)

The figures show estimated cost savings if TIRS were implemented instead of SIRS, based on best (deterministic) estimates for all parameters. They include the much lower costs of spraying and case investigations and the slightly higher costs of diagnosis and treatment under TIRS relative to SIRS. They are presented per additional local malaria case (a) and per additional death incurred (b), disaggregated by year, and for both years combined. In year one, when incidence was lower, cost savings with TIRS were greater than in year two, when incidence was higher.

#### (a) Net cost savings per additional local malaria case

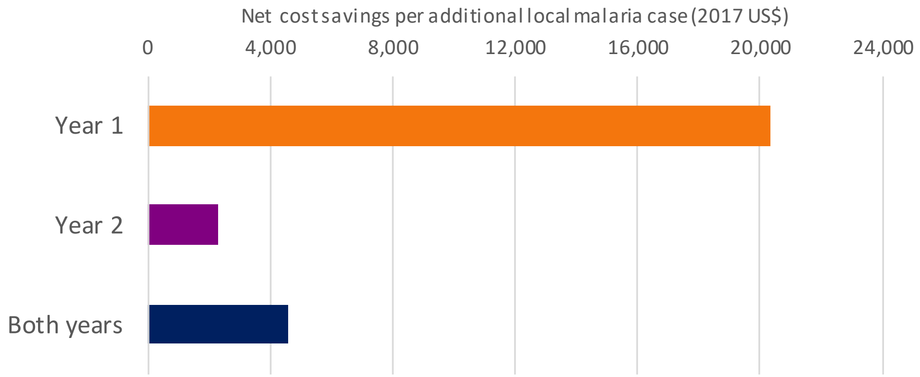


#### (b) Net cost savings per additional death incurred

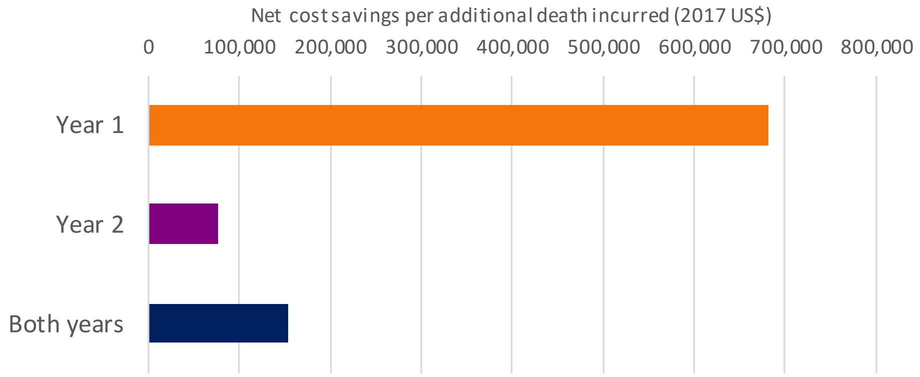

**S15: Restricted cost-effectiveness analysis – Mpumalanga only**

We conducted a restricted analysis over the two-year trial period using effectiveness data for Mpumalanga only (rather than the entire trial area), as follows:

- incidence rate (mean of cluster incidences) for SIRS (local cases per 1000py): 0.65 (95%CI 0.31-0.98); and
- rate ratio: 1.38 (95%CI 1.14-1.69).

Figure (a) shows the economic cost savings (health service perspective) and DALYs incurred by switching from SIRS to TIRS for the trial period. The large blue dot shows the mean incremental cost and mean incremental DALYs across the 10,000 model simulations. Individual model simulations are shown as smaller dots.

**(a) Cost-effectiveness plane (Mpumalanga only)**

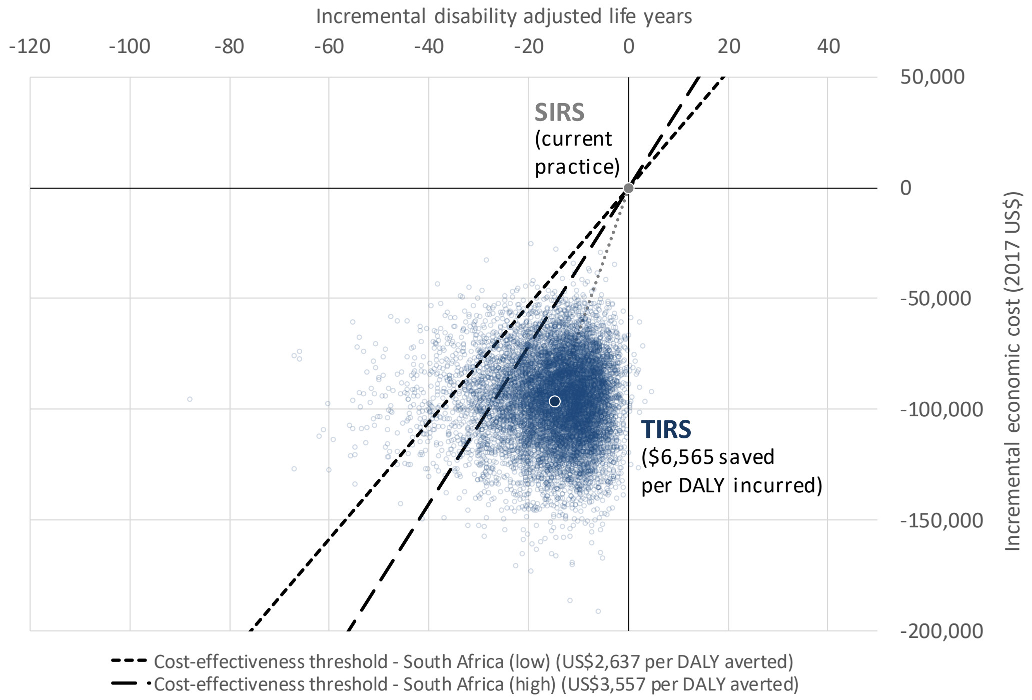

Figure (b) shows the probability that switching from standard IRS (SIRS) to targeted IRS (TIRS) is cost-effective at plausible cost-effectiveness thresholds over the two-year trial period.

**(b) Cost-effectiveness acceptability curve (Mpumalanga only)**

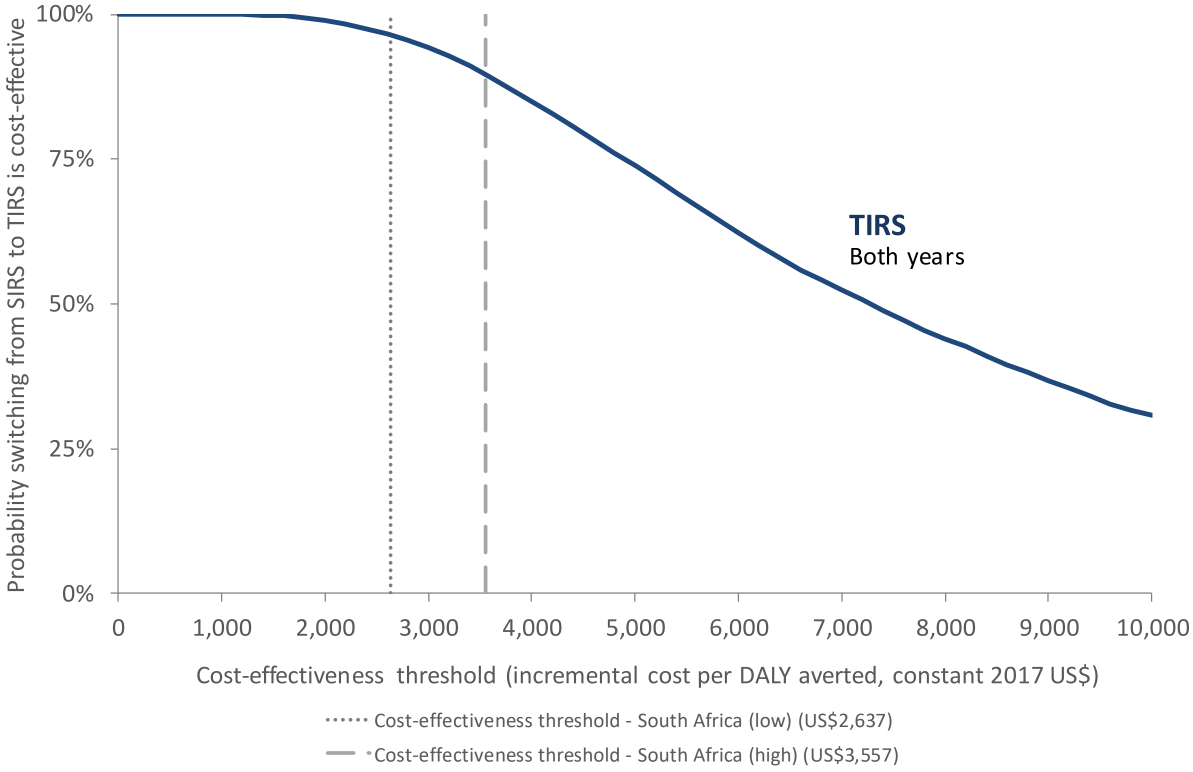

## References


1. Hoffmann TC, Glasziou PP, Boutron I, et al. Better reporting of interventions: template for intervention description and replication (TIDieR) checklist and guide. *BMJ* 2014; **348**: g1687.
2. Raman J, Allen E, Workman L, et al. Safety and tolerability of single low-dose primaquine in a low-intensity transmission area in South Africa: an open-label, randomized controlled trial. *Malar J* 2019; **18**(1): 209.
3. Wilkinson T, Sculpher MJ, Claxton K, et al. The International Decision Support Initiative Reference Case for Economic Evaluation: An Aid to Thought. *Value in health : the journal of the International Society for Pharmacoeconomics and Outcomes Research* 2016; **19**(8): 921-8.
4. Statistics South Africa. Community Survey 2016. Available from: https://municipalities.co.za/ (accessed 22 June 2020.
5. World Health Organization. World Malaria Report 2019. Geneva, 2019.
6. Salomon JA, Haagsma JA, Davis A, et al. Disability weights for the Global Burden of Disease 2013 study. *Lancet Glob Health* 2015; **3**(11): e712-23.
7. World Health Organization. Global Health Observatory data repository. Life-tables by country. https://apps.who.int/gho/data/node.main.LIFECOUNTRY (accessed 22 June 2020.
8. The Global Fund to Fight AIDS Tuberculosis and Malaria. Pooled Procurement Mechanism Reference Pricing: RDTs. https://www.theglobalfund.org/media/7564/psm_hivrdtreferencepricing_table_en.pdf (accessed 22 June 2020.
9. The Global Fund to Fight AIDS Tuberculosis and Malaria. Pooled Procurement Mechanism Reference Pricing: antimalarial medicines. . https://www.theglobalfund.org/media/5812/ppm_actreferencepricing_table_en.pdf?u=636784020480000000 (accessed 22 June 2020.
10. World Health Organization. Health service delivery costs. https://www.who.int/choice/cost-effectiveness/inputs/health_service/en/ (accessed 22 June 2020.
11. Turner HC, Lauer JA, Tran BX, Teerawattananon Y, Jit M. Adjusting for Inflation and Currency Changes Within Health Economic Studies. *Value in health : the journal of the International Society for Pharmacoeconomics and Outcomes Research* 2019; **22**(9): 1026-32.
12. World Bank. GDP deflator: linked series (base year varies by country) - United States, South Africa. https://data.worldbank.org/indicator/NY.GDP.DEFL.ZS.AD?end=2018&locations=US-ZA&start=2008&view=chart (accessed 22 June 2020.
13. World Bank. Official exchange rate (LCU per US$, period average) - South Africa.
